# Stressed target cancer cells drive nongenetic reprogramming of CAR T cells and solid tumor microenvironment

**DOI:** 10.1038/s41467-023-41282-x

**Published:** 2023-09-15

**Authors:** Yufeng Wang, David L. Drum, Ruochuan Sun, Yida Zhang, Feng Chen, Fengfei Sun, Emre Dal, Ling Yu, Jingyu Jia, Shahrzad Arya, Lin Jia, Song Fan, Steven J. Isakoff, Allison M. Kehlmann, Gianpietro Dotti, Fubao Liu, Hui Zheng, Cristina R. Ferrone, Alphonse G. Taghian, Albert B. DeLeo, Marco Ventin, Giulia Cattaneo, Yongxiang Li, Youssef Jounaidi, Peigen Huang, Cristina Maccalli, Hanyu Zhang, Cheng Wang, Jibing Yang, Genevieve M. Boland, Ruslan I. Sadreyev, LaiPing Wong, Soldano Ferrone, Xinhui Wang

**Affiliations:** 1 Division of Gastrointestinal and Oncologic Surgery, Department of Surgery, Massachusetts General Hospital, Harvard Medical School, Boston, MA USA; 2 Department of General Surgery, Tongji Hospital, School of Medicine, Tongji University, Shanghai, China; 3 Department of Gastrointestinal Surgery and General Surgery, First Affiliated Hospital of Anhui Medical University, Hefei, Anhui China; 4 Termeer Center for Targeted Therapies, Massachusetts General Hospital Cancer Center, Boston, MA USA; 5 Lineberger Comprehensive Cancer Center and Department of Microbiology and Immunology, University of North Carolina, Chapel Hill, NC USA; 6 Department of Hepatobiliary & Pancreatic Surgery and Liver Transplantation, Anhui Medical University, Hefei, Anhui China; 7 Biostatistics Center, Massachusetts General Hospital, Harvard Medical School, Boston, MA USA; 8 Department of Surgery, Cedars-Sinai Medical Center, Los Angeles, CA USA; 9 Department of Radiation Oncology, Massachusetts General Hospital, Harvard Medical School, Boston, MA USA; 10 Department of Anesthesia, Critical Care and Pain Medicine, Massachusetts General Hospital, Harvard Medical School, Boston, MA USA; 11 Research Department, Sidra Medicine, Doha, Qatar; 12 Vincent Center for Reproductive Biology, Vincent Department of Obstetrics and Gynecology, Massachusetts General Hospital, Harvard Medical School, Boston, MA USA; 13 Center for Comparative Medicine, Massachusetts General Hospital, Harvard Medical School, Boston, MA USA; 14 Department of Molecular Biology, Massachusetts General Hospital, Harvard Medical School, Boston, MA USA; 15 Department of Orthopaedics, Massachusetts General Hospital, Boston, MA USA

**Keywords:** Immunotherapy, Tumour immunology, Cancer microenvironment, Translational immunology, Cancer immunotherapy

## Abstract

The poor efficacy of chimeric antigen receptor T-cell therapy (CAR T) for solid tumors is due to insufficient CAR T cell tumor infiltration, in vivo expansion, persistence, and effector function, as well as exhaustion, intrinsic target antigen heterogeneity or antigen loss of target cancer cells, and immunosuppressive tumor microenvironment (TME). Here we describe a broadly applicable nongenetic approach that simultaneously addresses the multiple challenges of CAR T as a therapy for solid tumors. The approach reprograms CAR T cells by exposing them to stressed target cancer cells which have been exposed to the cell stress inducer disulfiram (DSF) and copper (Cu)(DSF/Cu) plus ionizing irradiation (IR). The reprogrammed CAR T cells acquire early memory-like characteristics, potent cytotoxicity, enhanced in vivo expansion, persistence, and decreased exhaustion. Tumors stressed by DSF/Cu and IR also reprogram and reverse the immunosuppressive TME in humanized mice. The reprogrammed CAR T cells, derived from peripheral blood mononuclear cells of healthy donors or metastatic female breast cancer patients, induce robust, sustained memory and curative anti-solid tumor responses in multiple xenograft mouse models, establishing proof of concept for empowering CAR T by stressing tumor as a promising therapy for solid tumors.

## Abstract (web summary)

The emergency of a high frequency of early memory T cells has been associated with clinical success of CAR T cell therapy. Here the authors show that target cancer cells stressed by disulfiram/copper complexes and ionizing radiation favour the reprogramming of CAR T cells that acquire memory-like characteristics, associated with prolonged anti-tumor response in preclinical solid tumor models.

## Introduction

Chimeric antigen receptor T-cell therapy (CAR T) has achieved unprecedented success as a novel immunotherapy with curative potential for certain hematologic cancers^1^. In contrast, results from clinical trials of CAR T for solid tumors have been disappointing^2,3^. Many factors contribute to the poor efficacy of CAR T for solid tumors. These include insufficient infiltration, expansion, persistence, and effector function, resulting in the ultimate exhaustion of adoptively transferred CAR T cells and an immunosuppressive tumor microenvironment (TME)^4^. In addition, intrinsic target antigen heterogeneity and/or antigen loss due to selective pressure by targeted therapies^5,6^ also contribute to the resistance of solid tumors to CAR T^7,8^. Significant efforts have been made to genetically engineer modified CAR T cells to promote more effective treatments for solid tumors. These include, but are not limited to, altering an array of tumor-specific CAR T cells to express (i) the p40 IL-23 subunit to promote proliferation and survival^9^, (ii) the dominant-negative TGF-β and adenosine receptor to promote proliferation^10,11^, (iii) the IL-8 receptor, CXCR1 or CXCR2, to enhance migration and persistence in the TME^12^, (iv) the anti-PD-L1 antibody, PD-1 dominant-negative receptor, or PD-1–knockout alteration to block PD-1/PD-L1 signaling in CAR T cells^13,14^, and (v) immunostimulatory RNA RN7SL1 to activate RIG-I/MDA5 signaling and promote expansion and effector-memory differentiation of CAR T cells. The cumulative effects of these alterations enhance myeloid cell and dendritic cell (DC) activity, reverse immunosuppressive TME conditions, and prime endogenous T cells to reject tumor cells with CAR-directed antigen loss^15^.

The percentage of CAR T cells with a central memory phenotype (T_CM_) is highly concordant with longer-term in vivo persistence and favorable clinical outcomes in neuroblastoma^16^. Moreover, stem-like memory T cells (T_SCM_) have been shown to play a critical role in mediating early anti-leukemic responses and long-term immune surveillance against leukemia relapse in patients for up to 3 years^17^. Importantly, sustained remission of chronic lymphocytic leukemia (CLL) and large B-cell lymphoma (LBCL) has been associated with elevated frequency of CD19 CAR T cells with memory-like characteristics^18,19^. More striking, the presence of an early memory T-cell population in the pre-manufacturing leukapheresis product predicted, with 100% accuracy, responders vs. non-responders to CD19 CAR T therapy among CLL patients^18^. These results underscore an essential requirement for successful CAR T, namely, a high frequency of early memory CAR T cells. However, genetically engineering one or even several genes produces a limited effect, and it is difficult to engineer a genetic solution that simultaneously addresses all or most of the existing barriers to treating solid tumors. Moreover, even though the efficacy of these genetically modified CAR T cells has been improved compared to the parental CAR T cells, they still yield low curative outcomes in preclinical models as well as in clinical trials for solid tumors^2^. Thus, we sought to develop a more broadly applicable, nongenetic approach that could overcome many, if not all, the obstacles that prevent CAR T from achieving long-lasting complete solid tumor rejection.

Disulfiram (DSF) is an irreversible pan-aldehyde dehydrogenase (ALDH) inhibitor approved by the FDA in 1951 for treating alcoholism^20^. DSF is also a chelator and primarily complexes with Cu^2+^ (DSF/Cu)^21^. It is well established that DSF/Cu targets the p97 segregase adaptor nuclear protein localization protein 4 (NPL4), which is essential for protein turnover and involved in multiple regulatory and stress-response signaling pathways^22^. By blocking NPL4/p97, DSF/Cu activates endoplasmic reticulum (ER) stress via upregulating the inositol-requiring enzyme 1 alpha (IRE1α)-X-box-binding protein 1 (XBP1) axis, leading to autophagic apoptosis^23^. Recently, we found that by combining ionizing radiation (IR) with DSF/Cu, we induced more robust immunogenic cell death (ICD) of differentiated cancer cells and cancer stem cells that couldn’t be achieved with either method alone^24^. The molecular characteristics of ICD include the release or cell-surface expression of highly immune-stimulatory damage-associated molecular pattern molecules (DAMPs). These molecules stimulate antigen-presenting cells (APCs), which boost CAR T expansion and activity^25^ and activate DCs, leading to subsequent development and activation of endogenous effector T cells and memory T cells^24,26^. In addition, DAMPs function as ligands for pattern recognition receptors (PRRs) and PRR agonists, which promote expansion and effector-memory differentiation of CAR T cells^15^. Therefore, we hypothesized that we could use DSF/Cu and IR-stressed target cancer cells to reprogram CAR T cells into early-memory T cells with more robust expansion, activation, persistence, and effector function, while simultaneously converting the immunosuppressive TME to an immunostimulatory TME by inducing the “hot death” (ICD) of cancer cells and release of pro-inflammatory cytokines and chemokines.

In this work, we investigate whether target cancer cells stressed by DSF/Cu and IR (DSF/Cu+IR) in vitro and in vivo could reprogram not only healthy donor or metastatic breast cancer patient donor-derived CAR T cells, but also the TME, thereby improving the efficacy of these effectors against solid tumors. This approach, which simultaneously addresses the multiple barriers to CAR T cell therapy, is assessed in vitro and in vivo by monitoring reprogrammed (RP) and non-reprogrammed (NRP) CAR T cells for evidence of early-memory T-phenotype switch, expansion, infiltration, effector function, and persistence; and by analyzing reprogrammed TME to identify the number of infiltrated immune cells, immune cell subtypes, cytokine/chemokine types, and levels. CAR T cells targeting different antigens and a panel of stressed solid tumors are evaluated for curative therapeutic potential using various established tumor cell lines and patient-derived xenograft (PDX)-derived solid tumor mouse models.

## Results

### DSF/Cu and IR induces cellular stress responses in target cancer cells

**Fig. 1:**
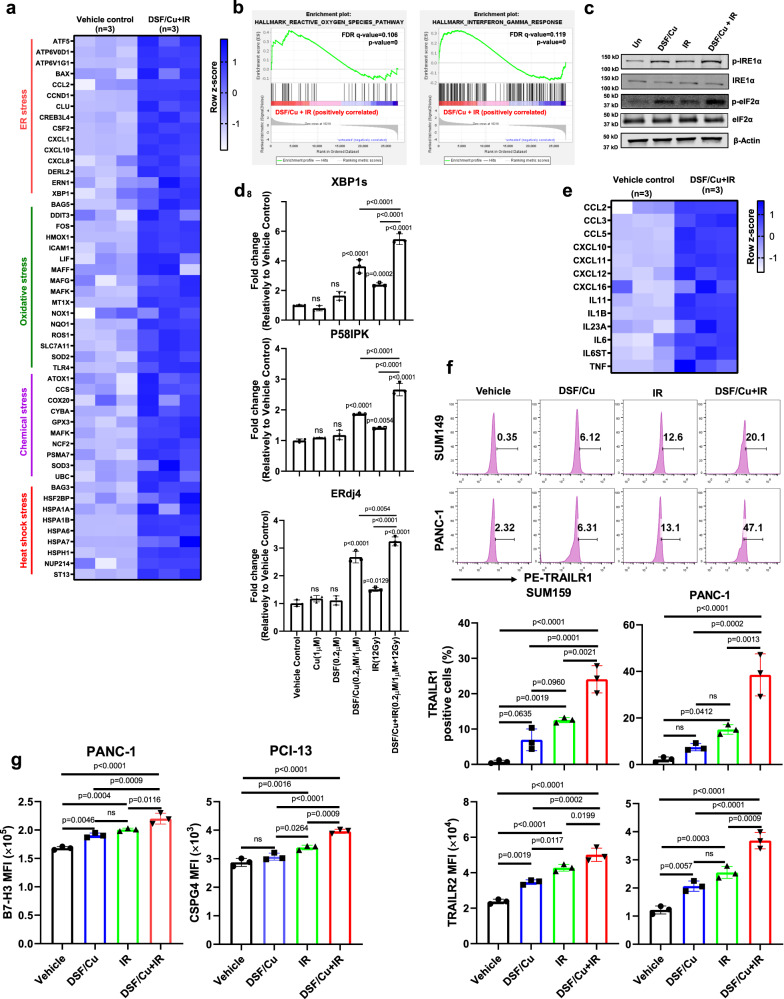
DSF/Cu + IR induces cellular stress responses in target cancer cells in vitro. **a** The heatmap shows ER stress, oxidative stress, chemical stress, and heat shock stress-related gene expression between vehicle-treated SUM159 tumor cells vs. DSF/Cu+IR-treated SUM159 tumor cells (*n* = 3 biologically independent experiments). **b** Representative GSEA enrichment plot illustrates the “INTERFERON_GAMMA_RESPONSE” and “REACTIVE_OXYGEN_SPECIES” (GSEA-computed *P*-values and false-discovery rate). **c** Western blot analysis of ER stress-related gene-encoded proteins IRE1α, p-IRE1α and eIF2α, p-eIF2α after indicated treatments (n = 3 independent experiments). **d** Detection by qRT-PCR of DSF/Cu+IR-induced ER stress indicator spliced XBP1(XBP1s) mRNA and its downstream target genes ERdj4, P58IPK in SUM159 cells. Fold-changes are shown as mean ± SD (*n* = 3 independent experiments). **e** Bulk cell RNA-seq shows the upregulation of pro-inflammatory chemokine and cytokine genes in DSF/Cu+IR-stressed SUM159 cells (*n* = 3 biologically independent experiments). **f** The percentage of TRAILR1 positive cells and mean fluorescence intensity (MFI) values of TRAILR2 (SUM149 and PANC-1) after indicated treatments (*n* = 3 independent experiments). **g** The MFI values of B7-H3/CSPG4 expression on PANC-1/PCI-13 cells 24 h post indicated treatments (*n* = 3 independent experiments). Statistical comparisons were performed using one-way ANOVA with Tukey’s multiple comparisons test (**d**, **f**, **g**). *P*-values are shown and error bars indicate mean ± SD. ns represents no significant difference. Source data are provided as a Source Data file.

Drawing on our previous work demonstrating that DSF/Cu at doses close to the half-maximal inhibitory concentration (IC50) of each cell line (i) activates ER stress, and (ii) combined with +IR(8 or 12 Gy) induces ICD by releasing membrane-bound and soluble factors that enhance immune cell functions^23,24^, we investigated a broad spectrum of stresses that can be induced by DSF/Cu (0.2 µM/1 µM) + IR(12 Gy) treatment of cancer cells. RNA-sequencing (RNA-seq) analysis revealed that genes related to ER, oxidative, chemical, and heat shock stress were upregulated in DSF/Cu+IR-treated vs. untreated target human breast cancer cells SUM159 (Fig. 1a). In addition, gene set enrichment analysis (GSEA) showed that genes of reactive oxygen species (ROS), interferon-gamma (IFN-γ) response, tumor necrosis factor (TNF-α), and inflammatory response pathways were enriched in DSF/Cu+IR-treated target cells (Fig. 1b and Supplementary Fig. 1a, b). Specifically, DSF/Cu+IR-induced ER stress in target cells was detected by increased p-eIF2a, p-IRE1α (Fig. 1c) activation of XBP1 axis (Fig. 1d and Supplementary Fig. 1c), and by membrane translocation of Erp57, calreticulin (CRT), and HSP90^3,23,27^ (Supplementary Fig. 1d). As a result, DSF/Cu+IR upregulated an array of pro-inflammatory chemokine and cytokine genes (Fig. 1e), increased cell membrane expression of tumor necrosis factor (TNF)-related apoptosis-inducing ligand receptor 1 (TRAILR1) and TRAILR2^28,29^ (Fig. 1f), and increased the CAR T cell targeted antigens, chondroitin sulfate proteoglycan 4 (CSPG4) and B7-H3, the tumor-associated antigens expressed by many tumor types, were chosen as targets for CAR T cells^30,31^ (Fig. 1g and Supplementary Fig. 2).

### DSF/Cu + IR-stressed cancer cells drive the phenotypic switch of CAR T cells with early memory-like characteristics

**Fig. 2:**
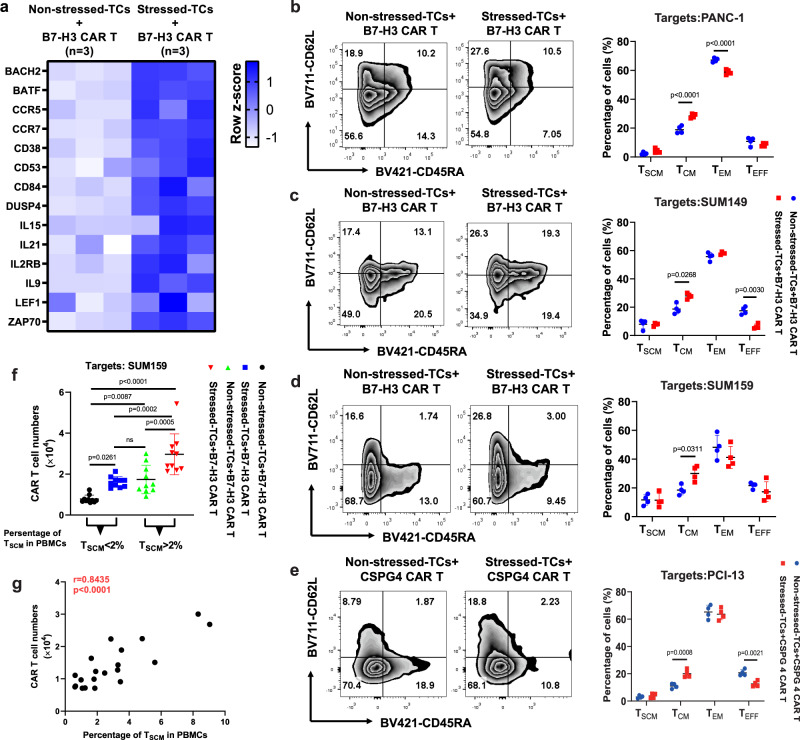
DSF/Cu + IR-stressed cancer cells drive phenotypic switch in CAR T cells with early memory-like characteristics. **a** The heatmap shows differences in memory T-cell-associated genes between B7-H3 CAR T cells co-cultured with non-stressed SUM159 tumor cells vs. DSF/Cu+IR-stressed cells (*n* = 3 biologically independent experiments). **b**–**e** Phenotypic analysis of CAR T cells with markers CD45RA and CD62L after in vitro reprogramming by 72 h co-cultured with target cells: SUM149 (**b**), SUM159 (**c**), PANC-1 (**d**) or PCI-13 (**e**). The frequencies of stem cell memory (T_SCM_, CD45RA^+^CD62L^+^), central memory (T_CM_, CD45RA^-^CD62L^+^), effector memory (T_EM_, CD45RA^-^CD62L^-^), and effector (T_EFF_, CD45RA^+^CD62L^-^) T cells in different groups are shown (*n* = 4 independent experiments). **f** The number of in vitro expanded CAR T cells from metastatic breast cancer patient-derived PBMCs show higher preexisting percentages of T_SCM_ (*n* = 10 individual donors) vs. lower percentages of T_SCM_ (*n* = 10 individual donors) after reprogramming by 72 h co-cultured with stressed vs. non-stressed target cells (*n* = 10 individual donors). **g** Positive correlation between in vitro expansion capacity of CAR T cells and preexisting percentage of T_SCM_ cells in PBMCs (*n* = 20 individual donors). Statistical comparisons were performed using two-way ANOVA with Sidak’s multiple comparisons test (**b**, **c**, **d**, **e**), one-way ANOVA with Tukey’s multiple comparisons test (**f**), and two-tailed Pearson’s *r* correlation (**g**). *P*-values are shown and error bars indicate mean ± SD. ns represents no significant difference. Source data are provided as a Source Data file.

Initially, we used RNA-seq analysis to assess changes in expression of memory-related genes in normal donor peripheral blood mononuclear cell (PBMC)-derived B7-H3 CAR T cells co-cultured with DSF/Cu+IR**-**stressed target cells [SUM159 cells were treated with DSF/Cu (0.2 µM/1 µM) (stressed) or DMSO (non-stressed) and cultured for 24 h, followed by 12 Gy IR]. Then the cells were immediately collected and washed twice with PBS and co-cultured with CAR T cells at E:T = 1:2 and compared to B7-H3 CAR T cells co-cultured with untreated target cells (Fig. 2a). Increased expression of memory-related genes, such as BACH2, BATF, CCR5, CCR7, IL-15, IL2RB, IL-9, LEF1, and ZAP70, was detected in CAR T cells co-cultured with stressed target cells. Next, owing in part to the transcriptome changes, we looked at the percentage of central-memory T cells (T_CM_), defined as CD45RA^-^CD62L^+^ cells, and found the percentage to be significantly higher in normal donor PBMC-derived B7-H3 CAR T cells following repetitive co-culture in separate experiments with three types of stressed cancer cells (stressed repetitive co-culture) compared with non-stressed cancer cells (regular repetitive co-culture) (Fig. 2b–d). A similar result was obtained with normal donor PBMC-derived CSPG4 CAR T cells following repetitive co-culture with stressed vs. non-stressed tumor cells (Fig. 2e). Next, we confirmed the ability of DSF/Cu+IR-stressed target cancer cells to drive the phenotypic switch of CAR T cells to an early-memory phenotype by examining B7-H3 CAR T cells derived from 20 breast cancer patients (Supplementary Table 1), which had been co-cultured with the stressed tumor cells. The percentage of preexisting stem-like memory T cells (T_SCM_), defined as CD45RA^+^CD62L^+^ cells, ranged from 0.61% to 9.04% in PBMCs from these 20 patients. To facilitate this analysis, we categorized the findings into two groups, i.e., low T_SCM_ in PBMCs (low T_SCM_/PBMC < 2%) and high T_SCM_ in PBMCs (high T_SCM_/PBMC > 2%). The rate of PBMC-derived CAR T cells with T_SCM_ phenotype was significantly higher in both low and high T_SCM_ groups following 48 h of co-culture with DSF/Cu+IR-stressed target breast cancer cells (SUM159) compared with B7-H3 CAR T cells co-cultured with untreated non-stressed SUM159 cells. Notably, stressed target cells drove the percentage of T_SCM_ within CAR T cells derived from the low T_SCM_/PBMC group to levels as high as the non-reprogrammed CAR T cells derived from the high T_SCM_/PBMC group (Fig. 2f).

These data indicate that our approach to generating CAR T cell preparations with an increased percentage of cells with early-memory-like characteristics, i.e., T_SCM_ or T_CM_ phenotype, may be worthy of being tested for converting clinical non-responders to responders and further enhancing antitumor response in responders to CAR T therapy. Finally, in agreement with the clinical findings in CLL and LBCL patients^18,19^, the levels of preexisting TSCM in PBMCs from the 20 breast cancer patients were highly correlated with the expansion capacity of their CAR T cells (Fig. 2g).

### DSF/Cu + IR-stressed target cells promote functional switch in CAR T cells with profoundly enhanced in vitro expansion and cytotoxicity

**Fig. 3:**
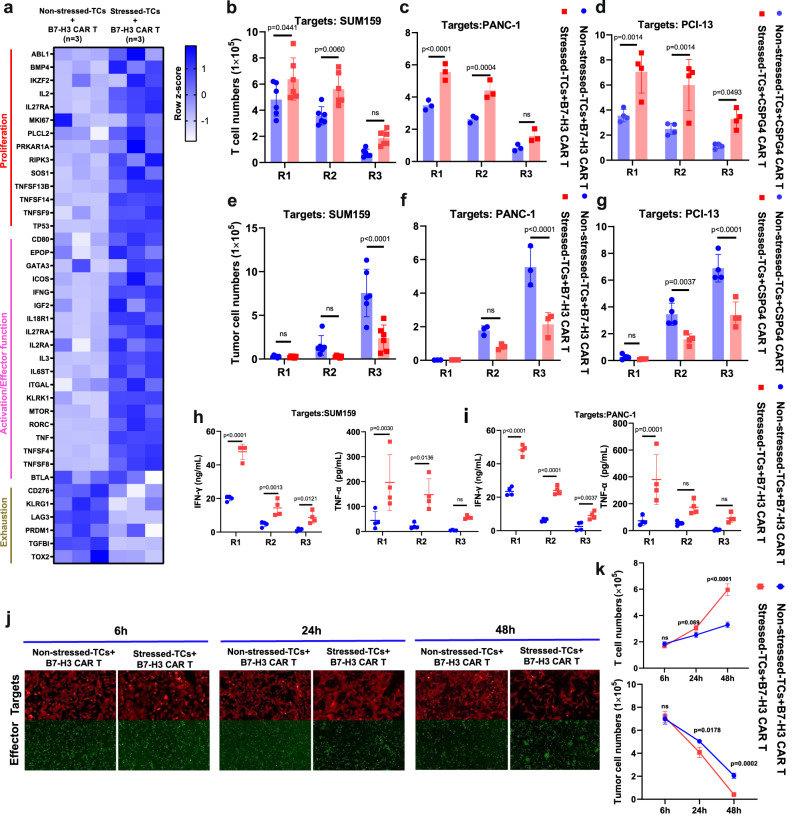
DSF/Cu + IR-stressed target cells promote functional switch in CAR T cells with profoundly enhanced in vitro expansion and cytotoxicity. **a** The heatmap shows differential gene expression between B7-H3 CAR T cells reprogrammed by co-culture with non-stressed SUM159 tumor cells vs. DSF/Cu+IR-stressed cancer cells (*n* = 3 biologically independent experiments). **b**, **c**, **d**, **e**, **f**, **g** The absolute number of CAR T (**b**, **c**, **d**) and tumor cells (**e**, **f**, **g**) was counted after each round of repetitive co-culture (E:T = 1:2) with non-stressed vs. DSF/Cu+IR-stressed cells: SUM159 (**b**, **e** *n* = 6 independent experiments), PANC-1 (**c**, **f** *n* = 3 independent experiments) or PCI-13 (**d**, **g** *n* = 4 independent experiments). **h**, **i** TNF-α and IFN-γ released in the supernatant collected at the end of each round of repetitive co-culture was measured by ELISA (*n* = 4 independent experiments). **j**, **k** Representative images (**j**) and quantitative (**k**, *n* = 3 independent experiments) data for CAR T cell proliferation and target cancer cell reduction after 6 h, 24 h, and 48 h of co-culture of target cancer cells with reprogrammed vs. non-reprogrammed CAR T cells. Statistical comparisons were performed using two-way ANOVA with Sidak’s multiple comparisons test (**b**, **c**, **d**, **e**, **f**, **g**, **h**, **i**, **k**). *P*-values are shown and error bars indicate mean ± SD. ns represents no significant difference. Source data are provided as a Source Data file.

Initially, we found higher levels of proliferation-related genes, such as IL2, IL27RA, MKI67, TNFSF13B, TNFSF14, TNFSF9, TP53, and T-cell activation genes, such as CD80, IFNG, MTOR, ICOS, KLRK1, RORC, TNFSF4, and TNFSF8, in normal donor PBMC-derived B7-H3 CAR T cells following 24 h of co-culture with DSF/Cu+IR**-**stressed target cells (Fig. 3a). Next, robust and sustained in vitro CAR T cell expansion was detected in normal donor PBMC-derived B7-H3 CAR T cells following stressed repetitive co-culture with two different breast cancer cell lines and two different pancreatic ductal adenocarcinoma (PDAC) cell lines compared to repetitive co-culture with non-stressed tumor cells (Fig. 3b, c and Supplementary Fig. 3a, b), as well as in normal donor PBMC-derived CSPG4 CAR T cells following repetitive co-culture with stressed vs. non-stressed *head and neck* squamous cell *carcinoma* (HNSCC) cell lines and triple negative breast cancer (TNBC) (Fig. 3d and Supplementary Fig. 3c). Then, strong and sustained cytotoxicity on a panel of different target cancer cells and increased release levels of IFN-γ and TNF-α were found in normal donor PBMC-derived B7-H3 CAR T cells or CSPG4 CAR T cells, respectively, following repetitive co-culture with stressed tumor cells (Fig. 3e–i and Supplementary Fig. 3d–f). Moreover, following co-culture with DSF/Cu+IR-stressed target cells (SUM159), the proliferation and cytotoxic activity of CAR T cells was shown to be time-dependent, i.e., detected as early as 6 h, and gradually became more apparent over 48 h as evidenced by the increased number of CAR T cells and fewer target cells (Fig. 3j, k).

Quantifying residual tumor cells and CAR T cells was performed by flow cytometry utilizing counting beads and staining for B7-H3 (expressed by tumor cells) and CD3 (expressed by CAR T cells). Finally, we speculated that upregulation of TRAIL on CAR T cells reprogrammed with DSF/Cu+IR-stressed tumor cells may have contributed to their enhanced target cell killing capacity^32^ (Supplementary Fig. 4).

### CAR T cells reprogrammed in vivo, using target cancer cells stressed by both intratumoral delivery of DSF/Cu and tumor localized IR, induced potent and sustained memory anti-solid tumor responses in multiple xenograft mouse models

**Fig. 4:**
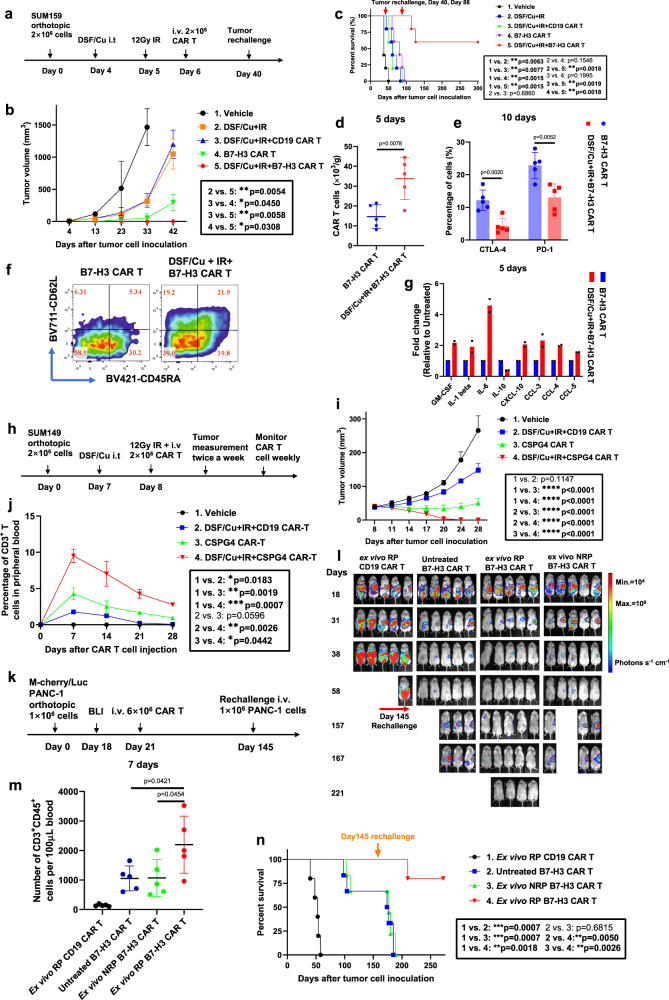
CAR T cells reprogrammed in vivo via target cancer cells stressed by intratumoral delivery of DSF/Cu and tumor localized IR induce potent, sustained, and memory anti-solid tumor responses in multiple xenograft mouse models. **a** Schema of the TNBC orthotopic xenograft model (SUM159) infused with CAR T cells. **b** Tumor volumes of each mouse/group (*n* = 5 mice/group) bearing SUM159 cell line-derived tumors. **c** Kaplan–Meier survival curve of mice (*n* = 5 mice/group). **d** Number of infiltrated CAR T (CD3^+^CD45^+^) in tumor 5 days after CAR T infusion. **e** Infiltrated CAR T expressing markers associated with T-cell exhaustion (CD3^+^CD45^+^CTLA-4^+^ or CD3^+^CD45^+^PD-1^+^) in tumors 10 days after CAR T infusion of each group (*n* = 5 mice/group). **f** Representative data of early memory CAR T, defined by detection of markers CD45RA and CD62L on CD3^+^CD45^+^ B7-H3 CAR T cells found in spleen 10 days after CAR T infusion. Percentages of T_SCM_/T_CM_/T_EM_/T_EFF_ are shown. **g** Cytokine and chemokine levels in tumors on day 5 after CAR T infusion (Tumor homogenate was pooled to yield 2 samples from 5 mice/group). **h** Schema of TNBC xenograft model (SUM149) infused with CAR T. **i** Tumor volumes of each mouse/group (*n* = 5 mice/group) in each group of mice bearing SUM149-derived tumors. **j** Percentage of CAR T cells (CD3^+^) in peripheral blood collected weekly from mice bearing SUM149-derived tumors (Blood was pooled to yield 3 samples from 5 mice/group). **k** Schema of orthotopic PDAC model (PANC-1) infused with CAR T. **l** Orthotopic PDAC tumor burden measured by BLI in each group treated as indicated (*n* = 5 mice/group). **m** Number of circulating CAR T (CD3^+^CD45^+^) on day 7 collected from mice bearing PDAC after T-cell injection of each group (*n* = 5 mice/group). **n** Kaplan–Meier survival curve of mice bearing PDAC in each group after tumor rechallenge (*n* = 5 mice/group). Statistical comparisons were performed using two-way ANOVA with Tukey’s multiple comparisons test (**b**, **i**, **j**), log-rank test (**c**, **n**), two-tailed unpaired *t*-test (**d**, **e**), and one-way ANOVA with Tukey’s multiple comparisons test (**m**). *P*-values are shown and error bars indicate mean ± SD. ns represents no significant difference. Source data are provided as a Source Data file.

Based on promising in vitro data, we performed in vivo tests of tumor-stressing via both intratumoral injection (i.t.) of DSF/Cu and IR to determine if we could enhance the antitumor efficacy of normal donor PBMC-derived CAR T cells in mice bearing human cancer cell line-derived xenografts (Fig. 4a). In the initial experiments, we treated tumors locally with DSF/Cu+IR to reprogram the systemically administered B7-H3 CAR T cells designated as RP-CAR T cells. This resulted in complete tumor regression in 100% of NSG mice bearing orthotopic TNBC SUM159 cell line-derived xenografts (Fig. 4b). Strikingly, when these mice were rechallenged on day 40 by inoculating SUM159 cells into the opposite lateral mammary fat pad, the tumors were gradually rejected after initially forming in all mice. In contrast, the primary and rechallenged tumors continued to grow in the B7-H3 CAR T cell (non-reprogrammed or NRP-B7-H3 CAR T cells), the DSF/Cu+IR, DSF/Cu+IR + CD19 CAR T cell, and in the vehicle-treated control groups of mice (Fig. 4b and Supplementary Fig. 5a–d). Moreover, DSF/Cu+IR + RP-B7-H3 CAR T cell-treated mice exhibited long-lasting, tumor-free survival.

To further test the strength and duration of the immunologic memory response, the RP-B7-H3CAR T cell-treated tumor-free mice were rechallenged for the second time on day 88 with SUM159 cells and 60% (3/5) of the mice rejected the challenge, maintaining long-lasting, tumor-free survival (Fig. 4c). To understand, at least part, the reason for this durable and potent memory antitumor response, we examined the phenotypes of CAR T cells and their infiltration into tumors of RP-B7-H3 CAR T cell-treated tumor xenograft mice. We found that RP-B7-H3 CAR T cell-treated mice had higher levels of splenic T_SCM_ (CD45RA^+^CD62L^+^), T_CM_ (CD45RA^-^CD62L^+^) and tumor-infiltrated CAR T cells (CD3^+^CD45^+^), in addition to having fewer CAR T cells expressing markers associated with T-cell exhaustion (CD3^+^CD45^+^CTLA-4^+^/PD-1^+^) and higher pro-inflammatory cytokines/chemokines in tumor tissues, compared to mice treated with B7-H3 CAR T cells only (Fig. 4d–g). Next, we tested whether the same strategy could be used in vivo to boost the anti-solid tumor efficacy of RP-CAR T cells targeting a different antigen, i.e., CSPG4 (Fig. 4h). Likewise, the reprogrammed CSPG4 CAR T cell (RP-CSPG4 CAR T cell) treatment caused complete tumor regression of orthotopic TNBC SUM149 cell line-derived solid tumors in 100% of mice. In contrast, CSPG4 CAR T or DSF/Cu+IR + CD19 CAR T treatment inhibited tumor growth, but neither treatment caused complete tumor regression (Fig. 4i). In vivo expansion and persistence of CSPG4 CAR T cells (CD3^+^CD45^+^) was significantly higher in mice treated with RP-CSPG4 CAR T cells compared to NRP-CSPG4 CAR T cells (Fig. 4j).

Last, we assessed whether the same strategy could boost the antitumor activity of B7-H3 CAR T cells in a PDAC xenograft model, namely, PDAC PANC-1 cell line-derived xenografts in mice (Supplementary Fig. 6a). Again, RP-B7-H3 CAR T treatment resulted in complete tumor regression in 100% of mice while NRP-B7-H3 CAR T treatment inhibited tumor growth significantly but did not cause complete tumor regression (Supplementary Fig. 6b). In vivo expansion and persistence of B7-H3 CAR T cells at early (day 8) or late (day 92) time points after CAR T infusion in each mouse group was positively reflected in their antitumor efficacy (Supplementary Fig. 6b–d). RP-B7-H3 CAR T-treated mice had long-lasting tumor-free survival (Supplementary Fig. 6e). To further confirm the strength and duration of the immunologic memory response that was previously observed in the SUM159 mouse model (Fig. 4a–f), all tumor-free mice were rechallenged on day 135 with PANC-1 cells; 60% (3/5) of mice rejected the tumor rechallenge and remained long-lasting tumor-free (Supplementary Fig. 6e). Subsequently, we asked the question, if there is no accessible tumor for IR and /or i.t. injection of DSF/Cu, could this approach be applied ex vivo to enhance CAR T cell antitumor efficacy? To this end, we determined in vivo the anti-solid tumor activity of magnetic bead-sorted B7-H3 CAR T cells following 48 h in vitro co-culture with PANC-1 cells pre-stressed by DSF/Cu and IR, designated as ex vivo reprogrammed (ex vivo RP) CAR T cells (Fig. 4k). Sorted B7-H3 CAR T cells following 48 h in vitro co-culture with untreated PANC-1 cells, designated ex vivo non-reprogrammed (ex vivo NRP) CAR T cells, and non-co-cultured (untreated) B7-H3 CAR T cells served as controls.

The ex vivo RP-B7-H3 CAR T cells mediated complete tumor rejection in 100% of mice bearing orthotopic PANC-1 cell line-derived solid tumor xenografts, and the mice remained tumor-free for the length of the experiment. In comparison, treatment with ex vivo NRP and untreated B7-H3 CAR T cells resulted in significant tumor growth inhibition or undetectable tumor by bioluminescence imaging (BLI) in only 40 and 60% of mice, respectively (Fig. 4l). In vivo expansion and persistence of B7-H3 CAR T cells was significantly higher in mice treated with ex vivo RP-B7-H3 CAR T cells compared to other groups (Fig. 4m). To assess whether ex vivo RP-B7-H3 CAR T cells could also induce an immunologic memory response equivalent to that obtained by i.t. injection of DSF/Cu and tumor localized IR, we rechallenged all mice with PANC-1 cells on day 145. A strong immunologic memory response was observed in 80% (4/5) of mice treated with ex vivo RP-B7-H3 CAR T cells, as measured by tumor rejection and/or remaining tumor-free, while 20% (1/5) developed tumors (Fig. 4n).

### Tumors stressed by DSF/Cu and IR reverse immunosuppressive TME in humanized mice

**Fig. 5:**
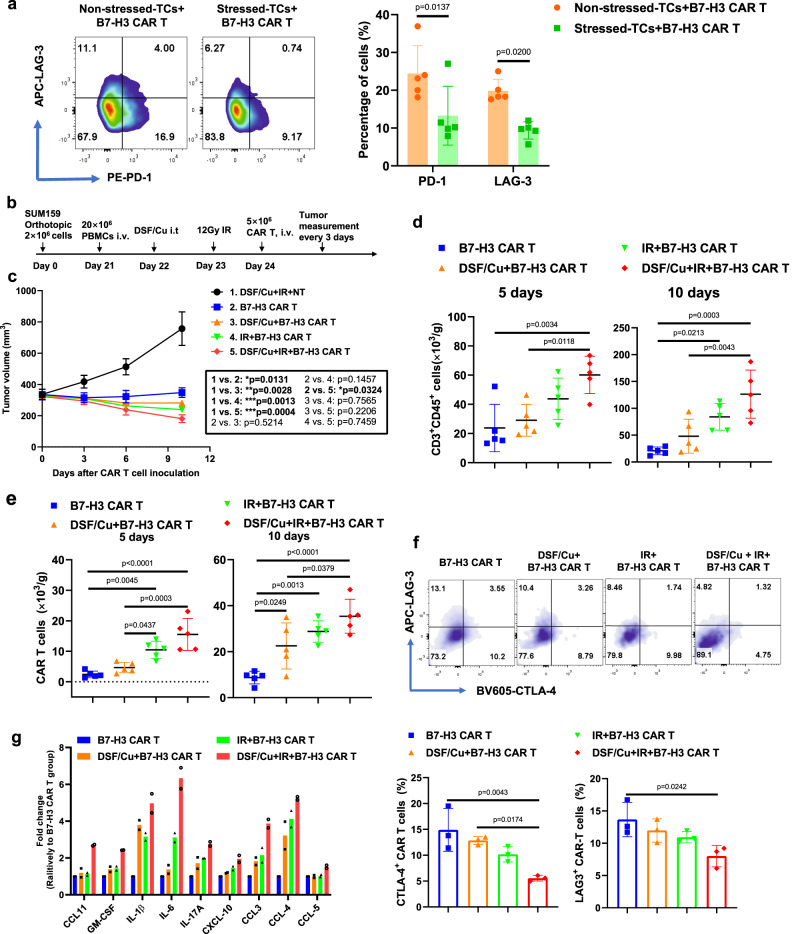
Tumors stressed by DSF/Cu and IR reverse immunosuppressive TME in humanized mice. **a** Exhausted B7-H3 CAR T cells (CD3^+^PD-1^+^ or CD3^+^LAG-3^+^) after round 3 (R3) of repetitive co-culture with DSF/Cu+IR-stressed vs. non-stressed PANC-1 cancer cells (*n* = 5 independent experiments). **b** Schema of the humanized mouse tumor model. **c** Tumor volumes (*n* = 5 mice/group) in the humanized mice were measured every 3 days. **d** Number of the total human CD3^+^ CD45 ^+^ cells including engrafted human PBMC and CAR T cells in tumor tissues 5 days and 10 days after CAR T cell infusion (*n* = 5 mice/group). **e** Number of B7-H3 CAR T cells in tumor tissues 5 days and 10 days after CAR T cell injection (*n* = 5 mice/group). **f** B7-H3 CAR T cells expressing markers associated with T-cell exhaustion (CD3^+^CD45^+^LAG-3^+^ or CD3^+^CD45^+^CTLA-4^+^) in tumor-infiltrating CAR T cells in tumor tissues 10 days after CAR T cell infusion (*n* = 5 mice/group). **g** Cytokine and chemokine levels in tumor tissues on day 5 after CAR T cell injection (Tumor homogenate was pooled to yield 2 samples from 5 mice/group). Statistical comparisons were performed using two-tailed unpaired *t*-test (**a**), two-way ANOVA with Tukey’s multiple comparisons test (**c**), and one-way ANOVA with Tukey’s multiple comparisons test (**d**, **e**, **f**). *P*-values are shown and error bars indicate mean ± SD. Source data are provided as a Source Data file.

Recently, we reported that DSF/Cu and IR-induced ICDs of breast cancer cells^24^ can promote antitumor immune responses. In this study, we found that DSF/Cu+IR upregulated an array of pro-inflammatory chemokine and cytokine genes (Fig. 1e), increased tumor infiltration of CAR T cells (Fig. 4d), and decreased levels of exhausted CAR T cells in vitro and in vivo (Figs. 4e and  5a; Supplementary Fig. 7a–h). To gain knowledge about the effect of our approach in immunocompetent mice, we established a humanized murine model by engrafting human immune cells in NSG mice (Supplementary Fig. 8) and investigated DSF/Cu+IR-stressed tumor-induced TME changes in humanized mice bearing TNBC SUM159 cell-derived xenografts (Fig. 5b). When the average tumor volume reached approximately 350 mm^3^, the DSF/Cu+IR + B7-H3 CAR T treatment was initiated. Owing to the rapid response to this therapy, the tumors shrank quickly in mice (Fig. 5c). To ensure a sufficient volume of tumor tissues would be available for subsequent analyses, specimens had to be collected on days 5 (*n* = 5 mice/group) and 10 (*n* = 5 mice/group) after CAR T cell infusion. At both time points, we found that DSF/Cu+IR + B7-H3 CAR T cell-treated mice had higher levels of infiltrated T cells (CD3^+^CD45^+^) (Fig. 5d), CAR T cells (Fig. 5e), as well as lower levels of CAR T cells expressing markers associated with T-cell exhaustion (LAG-3^+^CTLA-4^+^, for simplicity, referred to as exhausted-like or exhausted cells) in the TME (Fig. 5f) compared to those found in mice treated with either IR + B7-H3 CAR T or DSF/Cu+B7-H3 CAR T or B7-H3 CAR T. In agreement with the findings from the TME of NSG mice (Fig. 4g), a distinctive signature of upregulated pro-inflammatory cytokines and chemokines, including IL-1β, IL-6, IL-17A, GM-CSF, CCL11, CXCL-10, CCL3, CCL4 and CCL5, was detected at high magnitude protein levels in the TME of mice treated with DSF/Cu+IR + B7-H3 CAR T cells. In comparison, levels of the above distinctive signature of cytokines/chemokines were also upregulated but to a lesser extent in the TME of mice treated with IR + B7-H3 CAR T cells or DSF/Cu+B7-H3 CAR T cells (Fig. 5g). These pro-inflammatory cytokines and chemokines may partially account for the reprogramming initiated by DSF/Cu+IR, enabling the conversion of an immunosuppressive TME to an immune supportive TME^33,34^.

### Activation of the JAK/STAT signaling axis can be attributed to stressed cancer cell-induced phenotypic and functional switches of CAR T cells

Next, we sought to identify the main responder when CAR T cells encounter DSF/Cu+IR-stressed cancer cells. As indicated earlier, DSF/Cu+IR induces cell stress responses leading to upregulation of an array of pro-inflammatory chemokine and cytokine genes in target cancer cells (Fig. 1e). Of these, the following major changes attracted our attention: (i) genes leading to the activation of JAK/STAT signaling pathways (Supplementary Fig. 9a, b), (ii) time-dependent increases of IL-6 release, most noticeably detected in DSF/Cu+IR-stressed SUM159 cells (Supplementary Fig. 9c), as well as the predominant increase of IL-6 compared to other cytokines/chemokines in the TME of NSG or NSG-humanized mice treated with DSF/Cu+IR (Figs. 4g and  5g), and (iii) the profound increase of IFN-γ released by CAR T cells following stressed repetitive co-culture with 2 types of target cells (Fig. 3h, i). It is well established that IL-6 and IFN-γ are potent activators of the JAK/STAT pathway, which plays a pivotal role in cytokine receptor signaling and governance of T-cell phenotype, proliferation, survival, and function^35,36^. These data prompted proof of principle experiments to determine if the stressed cancer cell-induced CAR T cell phenotype and functional switches are truly dependent on the IL-6-JAK/STAT signaling axis. When CAR T cells were co-cultured with DSF/Cu+IR-stressed SUM159 cells, they showed a higher level of activation of the JAK/STAT axis than when co-cultured with untreated SUM159 cells. This was indicated by the upregulation in CAR T cells of phosphorylated (p)-JAK1, p-JAK2, p-STAT3, p-STAT5 and its target anti-apoptotic protein BCL-2 (Supplementary Fig. 9d). We then demonstrated that JAK inhibitors (JAKi), decreased T_CM%_ in B7-H3 CAR T cells in a dose-dependent manner (Supplementary Fig. 9e). More important, JAKi completely abolished, in a dose-dependent fashion, the effects of increasing T_CM%_ of B7-H3 CAR T cells by DSF/Cu+IR-stressed cancer cells (Supplementary Fig. 9e). However, JAKi ruxolitinib or momelotinib decreased the target cell in vitro killing capacity of B7-H3 CAR T cells, regardless of their co-culture with non-stressed or stressed tumor cells (Supplementary Fig. 9f). Nevertheless, the in vivo antitumor effect mediated by stressed tumor-reprogrammed CAR T/TME was completely abolished by ruxolitinib (Supplementary Fig. 9g).

### Robust and long-sustained therapeutic responses against solid tumors by RP-B7-H3 CAR T derived from PBMC of patients with metastatic breast cancer

**Fig. 6:**
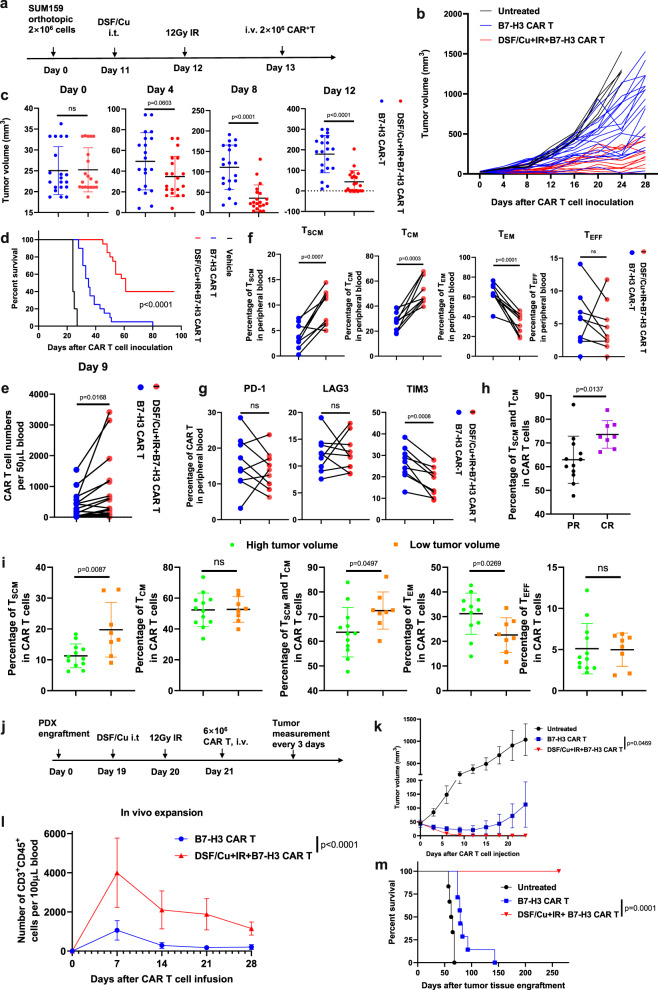
Robust and long-sustained therapeutic responses against solid tumors by RP B7-H3 CAR T derived from PBMCs of patients with metastatic breast cancer. **a** Schema of TNBC orthotopic xenograft model (SUM159) infused with CAR T. **b** Tumor volumes (untreated *n* = 5, CAR T *n* = 20, DSF/Cu+IR + CAR T *n* = 20) measured every 4 days. **c** Comparison of tumor volumes in mice treated with the same patient’s PBMC-derived CAR T vs. DSF/Cu+IR + CAR T on days 0, 4, 8, 12 post-CAR T infusion (CAR T *n* = 20, DSF/Cu+IR + CAR T *n* = 20). **d** Kaplan–Meier survival curve of mice (untreated *n* = 5, CAR T *n* = 20, DSF/Cu+IR + CAR T *n* = 20). **e** Frequency of B7-H3 CAR T cells in peripheral blood 9 days post-CAR T injection (CAR T n = 20, DSF/Cu+IR + CAR T *n* = 20). **f** Early memory B7-H3 CAR T, defined by CD45RA and CD62L on CD3^+^ in peripheral blood collected from mice 10 days after the CAR T infusion. Percentages of T_SCM_/T_CM_/T_EM_/T_EFF_ CAR T are shown (*n* = 9). **g** Frequency of B7-H3 CAR T expressing markers associated with T-cell exhaustion (CD3^+^PD-1^+^, CD3^+^LAG3^+^, or CD3^+^TIM3^+^ in peripheral blood collected from treated mice 9 days post CAR T injection (n = 9/group). **h** Comparison of percentages of circulating T_SCM_ and T_CM_ of B7-H3 CAR T in tumor-bearing mice with complete response (CR) vs. partial response (PR) 24 days post CAR T infusion (CR: *n* = 12, PR: *n* = 8). **i** Reverse correlation of percentages of early/effector-memory NRPB7-H3 CAR T with tumor burden (high tumor volume group *n* = 12; low tumor volume group *n* = 8). **j** Schema of TNBC-PDX mouse model. **k**, Tumor volumes (vehicle n = 6; the other two groups *n* = 7) of each group. **l** Frequency of B7-H3 CAR T cells (CD3^+^CD45^+^) in the blood of mice collected weekly (*n* = 7/group). **m** Kaplan–Meier survival curve of mice (vehicle *n* = 6; the other two groups *n* = 7). Data “*n*” represents biological replicates. Statistical comparisons were performed using a two-tailed unpaired *t*-test (**c**, **h**, **i**), log-rank test (**d**, **m**), two-tailed paired *t*-test (**e**, **f**, **g**), and two-way ANOVA with Tukey’s multiple comparisons test (**k**, **l**). *P*-values are shown and error bars indicate mean ± SD. Source data are provided as a Source Data file.

To corroborate the clinical relevance of our approach to enhance the ability of CAR T to eradicate solid tumors, the therapeutic efficacy of RP-CAR T cells derived from metastatic breast cancer patients with 1.01–9.51% T_SCM_ in PBMCs (Supplementary Data 1) was evaluated. RP-B7-H3 CAR T cells and NRP-B7-H3 CAR T cells derived from each patient (*n* = 20) were compared for therapeutic responses in the orthotopic TNBC SUM159 mouse model (Fig. 6a). RP-B7-H3 CAR T cells showed more robust therapeutic responses than NRP-B7-H3 CAR T cells derived from the same patients (Fig. 6b). Twelve days after CAR T cell infusion, complete tumor rejection was observed in mice treated with RP-B7-H3 CAR T from patients with metastatic breast cancer; 12 days after CAR T cell infusion, complete tumor rejection was found in 40% (8/20) of mice treated with RP-B7-H3 CAR T, while 5% (1/20) of the mice treated with NRP-B7-H3 CAR T cells derived from the same set of patients (Fig. 6c) exhibited complete tumor rejection. In summary, the 8 mice treated with RP-B7-H3 CAR T with complete tumor rejection remained tumor-free and healthy, while the other mice treated with NRP-B7-H3 CAR T, derived from the same PBMCs had shorter survival (Fig. 6d). It appears that the superior antitumor efficacy of RP-B7-H3 CAR T cells correlates with (i) increased CAR T cell expansion in vivo (Fig. 6e); (ii) more CAR T_SCM_ and CAR T_CM_ cells, together with fewer CAR T_EM_ and CAR T_EFF_ (Fig. 6f), and (iii) fewer TIM3^+^ exhausted CAR T cells (Fig. 6g).

Importantly, significantly more CAR T_SCM_ cells and CAR T_CM_ cells were detected in the peripheral blood of complete responder (CR) mice with complete tumor rejection than in partial responder (PR) mice with reduced tumor sizes in RP-CAR T cell-treated mice (Fig. 6h). Consistent with this finding, in NRP-B7-H3 CAR T cell-treated mice, significantly more CAR T_SCM_ cells and CAR T_CM_ cells were detected in peripheral blood of mice with low tumor burden than in mice with high tumor burden (Fig. 6i). Overall, the percentage of T_SCM_ in PBMCs and in CAR T cells favorably correlated with the in vivo antitumor response of these PBMC-derived CAR T cells (Supplementary Fig. 10a–e). Furthermore, B7-H3 CAR T cells derived from PBMC with low T_SCM%_ (0.85%), obtained from a 75-year-old metastatic TNBC patient, were tested in a TNBC PDX mouse model (Fig. 6j and Supplementary Fig. 11a). To this end, B7-H3^+^TNBC PDX tissue pieces were orthotopically engrafted into a group of NSG mice (Supplementary Fig. 11b). Subsequent RP-B7-H3 CAR T cell treatment resulted in complete tumor rejection in 100% of the treated mice. In sharp contrast, treatment with NRP-B7-H3 CAR T cells showed non-significant tumor growth inhibition compared to untreated mice (Fig. 6k). In vivo expansion and persistence of B7-H3 CAR T cells was significantly higher in mice treated with RP-B7-H3 CAR T cells than with NRP-B7-H3 CAR T cells derived from this patient (Fig. 6l). Most importantly, the RP-B7-H3 CAR T cell-treated mice remained tumor-free for the length of the experiment (Fig. 6m).

## Discussion

To overcome the therapeutic barriers of CAR T for solid tumors, genetically modified CAR constructs have been selectively developed to increase CAR T cell expansion, infiltration, function, and persistence as well as to decrease CAR T exhaustion and transform the immunosuppressive TME from a “cold” to “hot” state^4^. Given the challenges and limitations of genetically engineered CAR T for solid tumors, we bypassed this approach, focusing instead on nongenetic methods to stimulate or “stress” the targeted cancer cells. As a result of our investigation, we found that CAR T cells and TME could be significantly and beneficially reprogrammed by exposure to DSF/Cu+IR-stressed cancer cells. The cellular stresses induced by DSF/Cu+IR caused immunogenic cell death (ICD) or “hot death” of targeted cancer cells accompanied by the release of highly immune-stimulatory DAMPs, pro-inflammatory cytokines, and chemokines, which in turn, reprogram CAR T cells and TME^24^ (Fig. 1e). It is worth mentioning that when we investigated the impact on stressing tumor cells by each component alone or combined, i.e., DSF, Cu, DFS/Cu, IR, DSF/Cu+IR, we found DSF/Cu+IR is most effective in activating the XBP1 axis, a crucial parameter of ER stress (Fig. 1d and Supplementary Fig. 1c). It was reported that DSF alone activated mouse CD8^+^ T cells in a dose-dependent manner (1–10 µM)^37^. In contrast, DSF/Cu has a direct cytotoxic effect at a high dose (e.g., 1 µM/1 µM) on CAR T cells in vitro (Supplementary Fig. 12). Therefore, as demonstrated in this study, low DSF/Cu doses sufficient to induce stressing and ICD of tumor cells should be considered to reprogram CAR T cells and the TME.

The RP-CAR T cells were superior to CAR T cells in many of the key characteristics required for potent and sustained antitumor efficacy. For example, healthy donor PBMC-derived RP-CAR T cells compared to NRP-CAR T cells showed upregulated expression of memory T-cell-related genes (Fig. 2a) and higher percentages of T_CM_ (Fig. 2b–e) or T_SCM_ (Fig. 4f), leading to robust and sustained in vitro and in vivo CAR T cell expansion (Figs. 3b–d,  4j, and  6e; Supplementary Figs. 3a–c and  6c) and greater in vivo persistence^16^ (Fig. 6c,  m and Supplementary Fig. 6e). This finding is consistent with a recent clinical report that early memory T cells play a determinant role in the clinical success of CD19 CAR T cells in CLL^18^, which was further confirmed by the most recent long-term follow-up data from the phase III ZUMA-7 trial of CD19 CAR T cells in LBCL^19^. Second, RP-CAR T cells acquired higher levels of expression of proliferation-related and T-cell activation genes (Fig. 3a), greater cytotoxicity (Fig. 3e–k and Supplementary Fig. 3d–f), and increased release of IFN-γ and TNF-α (Fig. 3h, i). Third, the combination of healthy donor-derived CAR T cells and local treatment of tumors with DSF/Cu+IR resulted in the complete response of primary cell line or patient-derived TNBC or PDAC xenografts in 100% of treated mice (Figs. 4b, i and  6k; Supplementary Fig. 6b). Furthermore, as an indication of long and sustained immunological memory responses against tumors from the persistent residual CAR T cells, first-time rechallenged TNBC tumors were rejected in 100% of mice and PDAC tumors in 60% of mice (Fig. 4b and Supplementary Fig. 6e), while even second-time rechallenged TNBC tumors were rejected in 60% of mice (Fig. 4c). Finally, DSF/Cu+IR-induced ICD of cancer cells and the subsequent release of DAMPs, upregulated pro-inflammatory cytokines, and chemokines led to activated immune responses in the TME, in the absence of toxicity (Fig. 5g)^3,38^. Thus, the TME was reversed from “cold” to “hot”, as evidenced by the increased tumor infiltration of CAR T cells and decreased levels of exhausted CAR T cells in NSG mice (Fig. 4d, e). Similarly, elevated infiltrating T cells (CD3^+^CD45^+^) (Fig. 5d), CAR T cells (Fig. 5e), and lower expression of markers associated with T-cell exhaustion (LAG-3^+^CTLA-4^+^) were also observed in humanized mice (Fig. 5f).

In this study, we investigated the impact of in vivo reprogramming of B7-H3 CAR T cells via stressed tumor in TNBC-bearing mice by evaluating the efficacy of RP-B7-H3 CAR T cells derived from 20 patients with metastatic breast cancer, most of whom were heavily pretreated (Supplementary Data 1). RP-B7-H3 CAR T treatment resulted in 8/20 mice exhibiting a complete response (CR), accompanied by an increased number of early CAR T memory cells and fewer CAR T effector memory and exhausted CAR T cells, and in 12/20 mice exhibiting a partial response (PR), while NRP B7-H3 CAR T cell-treated mice exhibited 1/20 CR, accompanied by fewer CAR T early memory cells and increased CAR T effector-memory cells and exhaustion. These results establish the correlation between increased CAR T early memory cells in complete vs. partial responder mice and concur with the finding, as defined by the transcriptional profiles, that early vs. late memory CAR T cellular products from CLL are associated with a clinical response^18^. Most notably, when we treated mice bearing PDX with RP-B7-H3 CAR T cells derived from a metastatic TNBC patient, CR was obtained in 100% of mice, accompanied by increased CAR T cell in vivo expansion and long-lasting tumor-free survival. At the same time, compared to untreated mice, PR was observed with significant but diminished survival in mice treated with NRP-B7-H3 CAR T derived from the same patient. These data support further optimization of the in vivo reprogramming CAR T strategy to increase the ability to induce CR, spiking promise that *this type of* CAR T cell can offer the clinical benefit of CR for metastatic breast cancer patients.

Initially, our data indicated that activation of the JAK/STAT signaling axis was at least partially attributed to the reprogramming of CAR T cells by stressed cancer cells (Figs. 3h, i,  4g, and  5g; Supplementary Fig. 9a–d). This observation was further confirmed by activating the JAK/STAT pathway in CAR T cells. Co-culturing CAR T cells with stressed cancer cells and JAKi (ruxolitinib or momelotinib) completely abolished the DSF/Cu+IR-induced effects of increasing T_CM%_ (Supplementary Fig. 9e). Moreover, the antitumor effect mediated by stressed tumor-reprogrammed CAR T/TME was completely abolished by a JAKi (Supplementary Fig. 9g). Mechanistically, these data support the hypothesis that stressed cancer cells reprogram CAR T cells/TME is primarily through their upregulation of pro-inflammatory chemokines/cytokines which in turn activate the JAK/STAT pathway in CAR T cells. However, while JAKi inhibited the killing capacity of RP-CAR T cells, it also inhibited the killing capacity of NRP-CAR T cells (Supplementary Fig. 9f). Therefore, it appears that the mechanisms of reprogramming CAR T cells by stressed cancer cells are not limited solely to the activation of the JAK/STAT axis, which warrants further investigation.

Importantly, DSF/Cu+IR-stressed cancer cells not only reprogrammed CAR T cells and TME, but also became more sensitive to killing by CAR T cells as the result of ICD, higher expression of TRAILR1 and TRAILR2, and upregulated target antigens (Fig. 1c–f)^39^. It is also worth noting that ex vivo reprogrammed CAR T cells presented potent antitumor activity in vivo, like that observed in tumor-bearing mice treated directly with the combination of intratumoral DSF/Cu+IR and systemic CAR T (Fig. 4l–n). The advantage of ex vivo RP-CAR T is that this strategy can be used in cases where it is difficult to access the tumor for IR and/or i.t. injection of DSF/Cu. However, the overall effect of not having local delivery of DSF/Cu and IR, which induces stress on tumor cells and remodels the TME, remains unclear. A side-by-side study comparing both methods in terms of overall antitumor efficacy will be necessary to address this issue.

Notably, the data obtained from humanized mice demonstrate that DSF/Cu+IR-stressed cancer cells not only reprogram CAR T cells, but also enhance the infiltration of T and DC cells into tumors. These results imply that besides reprogramming CAR T cells, stressed cancer cells are highly likely to activate endogenous immune antitumor responses as the result of increased DAMPs, pro-inflammatory cytokines, and chemokines present within the TME. Furthermore, when the reprogrammed CAR T cells target B7-H3, an immune checkpoint protein, even more pronounced antitumor immune responses could be elicited^40^.

Therefore, this approach may simultaneously empower both passive and active immunity. Passive immunity in the host patient is mediated by the adoptive transfer of CAR T or T-cell receptor (TCR)-engineered T cells or tumor-infiltrating lymphocytes (TILs). Active immunity occurs when endogenous T cells recognize a variety of tumor antigens to prevent immune escape from CAR T- or TCR-engineered T-cell therapy aimed at one or a few tumor antigen target(s) which may be downregulated or lost^6^.

We have developed a nongenetic approach to reprogram and enhance the efficacy of CAR T cells and reverse the immunosuppressive TME by stimulating CAR T cells with DSF/Cu+IR-stressed target cancer cells. The reprogrammed CAR T cells acquire characteristics that promote therapeutic efficacy against solid tumors, including, but not limited to, robust cell expansion, long-term persistence, greater effector function, and decreased exhaustion. Intratumoral injection of DSF/Cu+ tumoral IR caused the release of elevated pro-inflammatory cytokines/chemokines, increased the infiltration of CAR T and T cells, and empowered the CAR T cells in the TME.

This approach overcomes most of the major obstacles that currently impede CAR T efficacy against solid tumors, yielding effectors that completely reject an array of distinct types of solid tumors, including TNBC, PDAC, and TNBC PDX, as observed in 100% of the mice tested in this study. In addition, the persistent residual RP-CAR T cells demonstrate long-term immunological memory responses as 60–100% of treated mice were protected from first-time tumor rechallenges, and 60% were protected from second-tumor rechallenges. Thus, stressing target cancer cells by DSF/Cu+IR is a timely approach for enhancing the efficacy of CAR T in solid tumors and inducing long-term immunological antitumor memory responses.

Currently, to enhance CAR T cell activity against solid tumors, there are active clinical trials investigating combinatorial approaches, such as oncolytic adenovirus combined with HER2-targted CAR T for multiple types of solid tumors (NCT03740256); pembrolizumab (PD-1 inhibitor) combined with EGFRvIII-targeted CAR T for glioblastoma (NCT03726515); PD-1 knockout engineered T cells combined with MUC1-targeted CAR T for advanced esophageal cancer (NCT03706326); varicella zoster virus combined with GD2-targted CAR T cells for advanced osteosarcoma and neuroblastoma (NCT01953900). In addition, immunomodulators, chemotherapy, radiation, immune checkpoint inhibitors or a virus vaccine have been combined with CAR T cells to improve their efficacy for hematological malignancies^41^. However, none of the clinical studies have explored a combination to reprogram CAR T and TME by stressing target cells. More importantly, DSF is an FDA-approved ALDH inhibitor for the treatment of alcoholism, and copper gluconate is a nutritional supplement. Both are repeatedly systemically delivered by oral administration (e.g., NCT02715609 and NCT04265274). As such, it is anticipated to be safe to intratumorally deliver DSF once (Antabuse dissolved in water)^22^ with water-soluble Cu at very low doses (e.g., DSF/Cu = 1.5 µM/1 µM), followed by a single dose of 12 Gy localized tumor radiation. The latter method was shown to be safe^42,43^. All these factors would greatly facilitate the clinical translation of our CAR T combinational approach to overcome multiple obstacles of CAR T for solid tumors.

## Methods

### Study approval

All mouse studies were performed under a protocol (#2019N000025) approved by the Institutional Animal Care and Use Committee (IACUC) at Massachusetts General Hospital. Blood collection was performed under a protocol (#13–416) approved by the Institutional Review Board (IRB) at Dana-Farber/Harvard Cancer Center.

### Cell lines and cell culture

Human TNBC cell lines, SUM149 and SUM159, were acquired from the Duke Comprehensive Cancer Center Cell Culture Facility and Asterand Bioscience, Inc., respectively. The human pancreatic ductal adenocarcinoma (PDAC) cell line PANC-1 was purchased from the American Type Culture Collection (ATCC). The head and neck squamous cell carcinoma (HNSCC) cell line PCI-13 was obtained from Dr. Theresa L. Whiteside’s Lab at UPMC Hillman Cancer Center, the University of Pittsburgh. The human PDAC cell line PDAC-6 was established at MGH using ascites fluid from a patient with metastatic PDAC^44^. In some cases, the cell lines were stably transduced with mCherry and firefly luciferase. The SUM149 and SUM159 cell lines were cultured in RPMI 1640 medium supplemented with 10% FBS. The PANC-1 and PDAC-6 cell lines were cultured in Dulbecco’s Modified Eagle’s Medium (DMEM) supplemented with 10% FBS. All cells were cultured at 37 °C in a 5% CO_2_ humidified atmosphere. All cell lines were authenticated by providers and tested negative for mycoplasma contamination.

### Antibodies

These antibodies were used for flow cytometry: R-Phycoerythrin AffiniPure F(ab’)_2_ Fragment Goat Anti-Mouse IgG (H + L) (cat#115-116–146, 1:100), Allophycocyanin (APC) AffiniPure F(ab’)_2_Fragment Goat Anti-Mouse IgG (H + L) (cat#115–136–146, 1:100), Fluorescein (FITC) AffiniPure F(ab’)_2_ Fragment Goat Anti-Mouse IgG (H + L) (cat# 115-096–146, 1:100) were obtained from Jackson ImmunoResearch. CD3-PE-Cy7 (cat#300420, clone: UCHT1, 1:100), CD8-APC-Cy7 (cat#344714, clone: SK1, 1:100), CD4-FITC (cat#357406, clone: RM4-5, 1:100), CD45RA- PE/Cyanine5 (cat#304110, clone: HI100, 1:100), CD45RO-APC (cat#304210, clone: UCHL1, 1:100), CD62L-PE (cat#304806, clone: MEL-14, 1:100), CD27-BV421 (cat#302824, clone: O323, 1:100), CD45RA-FITC (cat#304148, clone: HI100, 1:100), CD45RA-BV421 (cat#304130, clone: HI100, 1:100), PD-1 (CD279) -APC (cat#329907, clone: EH12.2H7, 1:100), PD-1 (CD279)-PE (cat#621608, clone: A17188B, 1:100), LAG-3 (CD223)-APC (cat#369212, clone: 7H2C65, 1:100), CCR7 (CD197) -BV421 (cat#353208, clone: G043H7, 1:100), CD261 (DR4, TRAIL-R1)-PE (cat#307206, clone: DJR1, 1:100), CD262 (DR5, TRAIL-R2)-APC (cat#307408, clone: DJR2-4 (7-8), 1:100), CD95 (Fas)-BV785 (cat#305646, clone: DX2, 1:100), CD127 (IL-7Rα)-PE (cat#351304, clone: A019D5, 1:100), TNFα-PE (cat#502909, clone: Mab11, 1:100), IFNγ-APC (cat#502512, clone: 4 S.B3, 1:100), Granzyme B-Pacific Blue (cat#515408, clone: GB11, 1:100), CD62L-BV711 (cat#304860, clone: DREG-56, 1:100), CD253 (TRAIL)-PE (cat#308206, clone: RIK-2, 1:100), CD45-PE (cat#368510, clone: 2D1, 1:100), CD366 (TIM3)-Pacific Blue (cat#345042, clone: F38-2E2, 1:100), CD279 (PD-1)-PerCP/Cyanine5.5 (cat#367410, clone: NAT105, 1:100), CD152 (CTLA-4)-BV605 (cat#369610, clone: BNI3, 1:100), CD25-PE/Dazzle™ 594 (cat#302646, clone: BC96, 1:100), CD276 (B7-H3)-APC (cat#351005, clone: MIH42, 1:100), CD11b-BV510 (cat#301334, clone: ICRF44, 1:100), CD14-APC/Cyanine7 (cat#325620, clone: HCD14, 1:100), CD33-PE/Cyanine7 (cat#366618, clone: P67.6, 1:100), CD86- PE/Cyanine7 (cat#374209, clone: BU63, 1:100), CD56-APC (cat#362503, clone: 5.1H11, 1:100), CD20-BV510 (cat#302339, clone: 2H7, 1:100), HLA-DR-APC (cat# 307609, clone: L243, 1:100), and Zombie Red™ Fixable Viability Kit (cat# 423110) were purchased from Biolegend. CD3-FITC (cat#555339, clone: HIT3a, 1:100), and FITC-Labeled Human B7-H3 (4Ig)/B7-H3b Protein (cat# B7B-HF2E7-25 µg, 0.5 µg/100 µL) were obtained from BD Biosciences and Acro Biosytems, respectively. These antibodies were used for western blot:ATF-6 (cat#65880S, 1:1000), Phospho-eIF2α (Ser51) (cat#3398, 1:1000), Bcl-2 (cat#15071S, 1:1000), β-Actin (#3700, 1:2000), STAT5 (cat#25656S, 1:1000), Phospho-Stat5 (Tyr694) (cat#9356S, 1:1000), STAT3 (cat#9139S, 1:1000), JAK1 (cat#3344S, 1:1000), Phospho-Jak2 (Tyr1007/1008) (cat#3771, 1:1000), Phospho-Stat3 (Tyr705) (cat#9145, 1:1000), Phospho-Jak1(Tyr1034/1035) (cat#74129, 1:1000) and Jak2 (cat#3230, 1:1000) were obtained from Cell Signaling Technology. Phospho-IRE1 alpha (Ser724) (cat# PA585647, 1:1000) was obtained from Invitrogen. These antibodies were used for CAR T generation: CD28 (cat#556620, clone: CD28.2, 1 µg/mL) were purchased from Biosciences, and CD3 (cat#130-093-387, clone: OKT3, 1 µg/mL) from Miltenyi Biotec.

### Animals

The 6-to-8-week-old NSG mice were obtained from the Massachusetts General Hospital (MGH) COX7 animal facility or Jackson laboratory. NSG mice were housed in autoclaved polysulfone individually ventilated cages (Allentown Caging, Allentown, NJ) in a specific pathogen-free (SPF) environment. Room lights were maintained on a 12:12-hour light: dark cycle. Room temperature was maintained at 68 to 71 F, and room humidity remained between 30% and 60%.

### In vitro stressing of target cells

Tumor cells were plated in 6-well culture plates at a density of 3 × 10^5^ cells/well (SUM149, SUM159) or 4 × 10^5^ cells/well (PANC-1, PDAC-6) in 2 mL RPMI 1640 medium containing 10% FBS and permitted to grow overnight. The following day, DSF/Cu (0.2 µM/1 µM) or DMSO was added and cultured for 24 h, followed by 12 Gy IR. The cells were then resuspended, washed twice with PBS, and set up immediately in 6-well plates at a density of 5 × 10^5^ cells/well. After 6–12 h, the tumor cells were co-cultured with CAR T cells at different E:T ratios.

### IR treatment in vitro and in vivo

For the in vitro treatment, cells were irradiated with a single dose of IR (12 Gray (Gy)). For tumors treated in vivo, one 12 Gy fraction was delivered locally to each mouse over the area of the tumor, while the remaining body was shielded with a lead drape. The X-RAD 320 Biological Irradiator (Precision X-ray Inc., CT) was used for these experiments.

### RNA extraction

Total RNA was extracted using RNeasy Mini Kit (Qiagen) according to the manufacturer’s instructions.

### Bulk RNA-seq and GSEA analysis

The total RNA content of both tumor cells and CAR T cells was extracted as described above, and the library was prepared by LC Sciences company. Raw data from both tumor cells and CAR T cells was normalized in three replicates for GSEA analysis. GSEA was performed using the GSEA_4.1.0 software (Broad Institute) on genes that differentially expressed on non-stressed vs. stressed-SUM159 cells; or NRP B7-H3 CAR T cells vs. RP B7-H3 CAR T cells. Genes used for drawing heatmaps were selected based on related publications^45,46^, (CCL-2 and CXCL-10)^47^, (CXCL1)^48^, (CXCL8)^49^, and the gene sets from The Molecular Signature Database (MSiDB) (Supplementary Table 2).

### Preparation of cell lysate from tumor tissue and cell culture

The tumor tissue was dissected and washed briefly with chilled PBS. The samples were placed in vessels containing lysis buffer, RIPA buffer (Thermo Fisher Scientific), and 1/50 (vol./vol.) of protease inhibitor (Thermo Fisher Scientific), and then disrupted for 30 s in the homogenizer. Tumor homogenates (2 µg/µL) were used for quantification of chemokines/cytokines. The cultured cells were detached and washed briefly with cold PBS before adding the lysis buffer. All samples were placed on ice for 30 min and then centrifuged for 20 min to collect the supernatant without disturbing the debris. The cell lysates were used for the western blot. All protein concentrations were quantified using a Pierce BCA Protein Assay Kit (Thermo Fisher Scientific) according to the manufacturer’s instructions.

### Western blotting

A total amount of 20 µg protein was loaded into 10% PAGE gel followed by electrophoresis and transferring to PVDF membranes, respectively. The membranes were blocked in 5% non-fat milk/TBST (Boston Bioproducts) for 1–2 h at room temperature and incubated with primary antibodies in 2% BSA and 5% non-fat milk in TBST at 4 °C overnight, washed 3 times using TBST and incubated with a corresponding secondary antibody diluted in the same buffer used for primary antibodies for 1 h at room temperature. Western blot data were collected using ODYSSEY Infrared imaging system Application software (VERSION 3.0). Blotting images were acquired with the blot scanner (LI-COR). Western Blot data were analyzed by Image Studio Lite (VERSION 5.2).

### Real-time quantitative reverse transcription PCR (qRT-PCR)

One microgram of total RNA extracted from cells was reverse transcribed using M-MLV reverse transcriptase purchased from Life Technologies (Carlsbad, CA, USA), according to the manufacturer’s instructions. The purity and concentration of RNA were estimated using Nanodrop 2000 (Thermo Scientific). The resulting cDNA was diluted to a concentration of 100 ng/μL in nuclease-free water. qRT-PCR using cDNA as a template with SYBR green detection was performed using a LightCycler 96 software (VERSION 1.1) with FastStart SYBR Green Master Mix purchased from Roche (Penzburg, Germany). The following real-time PCR primers (forward and reverse) were used: spliced XBP1s, 5’-TGCTGAGTCCGCAGCAGGTG-3’ and 5’-GCTGGCAGGCTCTGGGGAAG-3’; ERdj4, 5’-TCTTAGGTGTGCCAAAATCGG-3’ and 5’-TGTCAGGGTGGTACTTCATGG-3’; P58IPK, 5’-GGCTCGGTATTCCCCTTCCT-3’ and 5’-AGTAGCCCTCCGATAATAAGCAA-3’; 18S rRNA: 5′-TGTGCCGCTAGAGGTGAAATT-3′; 5′-TGGCAAATGCTTTCGCTTT-3′^50^.Each gene expression level was normalized to mRNA levels of the housekeeping gene 18S rRNA. Fold-changes of each gene’s expression were compared to that of the control sample using the 2^–ΔΔCT^ method.

### Flow cytometry

For cell-surface staining, the cells were harvested, washed twice with PBS, and then incubated with an appropriate amount of fluorochrome-conjugated or unconjugated antibodies for 20 min or 60 min at 4 °C in 2% PBB (2% BSA in PBS buffer). Fcγ fragment-specific secondary antibodies were used accordingly, if necessary. For intracellular staining, the surface-stained cells were fixed and permeabilized in Fixation/Permeabilization solution (BD Biosciences) for 20 min at 4 °C followed by two washings with Perm/Wash buffer (BD Biosciences). After intracellular staining was performed, the cells were incubated for 30 min at 4 °C in Perm/Wash buffer followed by two more washings with Perm/Wash buffer. To measure the number of CAR T cells that had infiltrated tumor tissues and spleens, the tumor samples were digested with collagenase IV (0.5 mg/mL; Sigma-Aldrich) and deoxyribonuclease (DNase) I (0.2 mg/mL; Sigma-Aldrich) for 30 min at 37 °C. Tumor digests and spleens were filtered through 70 µm cell strainers to obtain a single-cell suspension. ACK lysing buffer (Thermo Fisher) was used to lyse the red blood cells according to the manufacturer’s instructions. Viability dye was used in each sample. The cells were analyzed by flow cytometry using BD FACSDiva software (VERSION 8.0) or BD Accuri C6 software (VERSION 1.0.264.21) (BD Biosciences). The data were analyzed with FlowJo software (VERSION 10.8.1, Ashland, OR).

### Generation of CAR T cells

Peripheral blood mononuclear cells (PBMCs) were isolated from normal human donor blood (Research Blood Components, MA) or from patients (Supplementary Table 1 and Supplementary Data 1) with Lymphoprep (Stem cell Technologies). On day 0, the PBMCs (1 × 10^6^/well) were activated in a non-treated 24-well cell culture plate (#351147, Corning) pre-coated with 1 μg/mL CD3 (Miltenyi Biotec) and 1 μg/mL CD28 antibodies (BD Biosciences) in the complete medium (45% RPMI1640 and 45% Click’s medium [Irvine Scientific, CA], 10% FBS, 1% Penicillin and 1% Streptomycin [Corning]). On day 1, activated T cells were expanded by the addition of IL-7 (10 ng/mL, PeproTech, NJ) and IL-15 (5 ng/mL, PeproTech) (CAR T medium). On day 2, the activated and expanded T cells were transferred to wells of 24-well plates that had been previously coated with RetroNectin (Takara Bio Inc., Shiga, Japan) and contained retroviral particles of the CAR construct. On day 4, to allow for their continued expansion, the transduced cells were collected and transferred to tissue culture-treated 24-well plates (Cat#353047, Corning) with each well containing 0.5 mL of the activated T-cell suspension (5 × 10^5^ cells/well) and 1.5 mL of fresh CAR T medium. On day 6, an aliquot of transduced cells was analyzed for transduction efficiency. On day 8, CAR T cells were counted and reseeded at 1 × 10^6^/well in 2 mL of fresh CAR T medium to further expand cells. The medium was changed every 3 days. On days 12-13, CAR T cells grown at similar conditions were collected, aliquoted, and frozen for storage in a liquid nitrogen freezer for in vitro and in vivo experiments. Only blood from females was used since breast cancer is more prevalent in females than in males. Prior to collecting patient blood, informed consent was obtained by participants for both sample collection and the publication of clinical information that could potentially identify individuals.

### In vitro co-culture experiments

The stressed target cells were resuspended, washed with PBS twice, and set up immediately in 6-well culture plates at a density of 5 × 10^5^ cells/well (SUM149, SUM159, PDAC-6, PCI-13) or 7.5 × 10^6^ cells/well (PANC-1) in 2 mL complete CAR T medium^40^. After 6–12 h, the tumor cells were co-cultured with CAR T cells at the indicated E:T ratios.

### Repetitive co-culture assay

The tumor cells (untreated, DSF/Cu+IR-stressed) were seeded in 6-well plates at a density of 5 × 10^5^ cells/well (SUM149, SUM159, PDAC-6, PCI-13) or 7.5 × 10^6^ cells/well (PANC-1) in 2 mL of complete CAR T medium and grown overnight. Then, 2.5 × 10^5^ CAR T cells (*E*:*T* = 1:3 or 1:2) were added to the tumor cells. The CAR T cells eliminated 100% of the tumor cells within 2–5 days of co-culture (Round 1, R1). Next, the CAR T cells were collected and resuspended in 2 mL complete CAR T medium and transferred to wells containing either 5 × 10^5^ (SUM149, SUM159, PDAC-6, PCI-13) or 7.5 × 10^5^ (PANC-1) untreated tumor cells that had been seeded 12–14 h previously, and the co-culture was continued for 3 days. (Round 2, R2). The procedure was repeated once more (Round 3, R3), and the cells remaining from R3 were then collected for analysis of exhaustion-related markers (PD-1, LAG-3, TIM3).

### Phenotypic analysis of CAR T cells

To evaluate the phenotypic transformation of CAR T cells in vitro, cells from R1 of the repetitive co-culture assay were resuspended and washed twice with PBS. Thereafter, the cells were cultured in 2 mL complete CAR T medium supplemented with 5 ng/mL IL-15 and 10 ng/mL IL-7. After 3 days, the cells were collected and stained with CD3, CD4, CD8, CD62L, and CD45RA antibodies and then analyzed using flow cytometry.

### In vitro cytotoxicity and expansion of CAR T cells

Cells collected at the end of the co-culture assays were stained to quantify tumor cells with B7-H3-specific mAb 376.96 and CAR T cells with CD3-specific antibody. Counting beads (Thermo Fisher Scientific) were used based on the manufacturer’s instructions to obtain the absolute numbers of both residue tumor cells and CAR T cells.

### Enzyme-linked immunoassay (ELISA)

Cell culture supernatants (undiluted) were quantified for secreted cytokines (TNF-α, IFN-γ) according to the manufacturer’s instructions using ELISA kit (Biolegend). ELISA data were collected using All-In-One Microplate Reader Software Gen5 (VERSION 2.09).

### Isolation of reprogrammed CAR T cells

CAR T cells were co-cultured with DSF/Cu and IR-stressed tumor cells for 48 h to allow for reprogramming. The CAR T cells were subsequently isolated from the co-cultures as CD3^+^ T-cell Dynabeads (catalog# 11033, Themo Fisher) via positive selection. The magnetic bead isolated CAR T cells were used in Western blotting or ex vivo experiments in mice.

### Humanized mouse model

Human PBMCs (20 × 10^6^/per mouse) were engrafted into xenograft tumor-bearing or naïve 8–10 week-old female NSG mice via tail vein injection. The engrafted various types of immune cells were analyzed. Briefly, 500 µL of peripheral blood was collected from each mouse and pooled from all 5 mice on day 8 after humanization; red blood cells were removed using ACK lysing buffer, and the PBMCs were washed twice with PBS containing 0.5%BSA and stained with antibodies recognizing human CD3, CD4, and CD8, CD14, CD11b, HLA-DR, CD86, CD56, CD20. Then the samples were analyzed by the LSR II cytometer (BD Biosciences).

### In vivo solid tumor mouse models

Five distinct xenograft models were used in this study:

(i) TNBC cell line-derived orthotopic mouse model: SUM149 (2 × 10^6^) or SUM159 (2 × 10^6^) tumor cells in 100 µL FBS-free RPMI medium were injected into the mammary fat pads of 6-to-8-week-old female NSG mice. When the tumors became palpable, the mice were divided into groups using a stratified randomization strategy (*n* = 5 mice/group) and treated as indicated.

(ii) PDAC xenograft mouse model: PANC-1 (2 × 10^6^) tumor cells in 100 µL FBS-free RPMI medium were injected subcutaneously (s.c.) into the right thigh of 8-to-9-week-old male NSG mice. When the tumors became palpable, the mice were divided into groups using a stratified randomization strategy (*n* = 5 mice/group) and treated as indicated.

(iii) PDAC orthotopic xenograft mouse model: mCherry/Luc-PANC-1 (2 × 10^6^) tumor cells were orthotopically engrafted into the pancreas of 8-to-9-week-old male NSG mice. Tumors were measured by bioluminescence imaging (BLI) and divided into groups using a stratified randomization strategy (*n* = 5 mice/group). Bioluminescence imaging for animal study was collected using Perkin-Elmer IVIS100 imaging system (MA, USA). BLI imaging from animal studies were analyzed using Aura imaging software (VERSION 3.2).

(iv) Humanized mouse bearing TNBC orthotopic tumor model: SUM159 (2 × 10^6^) tumor cells in 100 µL FBS-free RPMI medium were injected into the mammary fat pads of 6-to-8-week-old female NSG mice. After 21 days, the mice were divided into groups using a stratified randomization strategy (*n* = 10 mice/group) and humanized by i.v. injection of 20 × 10^6^ human PBMCs per mouse from a heathy donor, and the mice were treated with the same donor-derived CAR T as indicated.

(v) TNBC-PDX orthotopic mouse model (Model ID: TM00096, The Jackson Laboratory, 1^st^ passage): A 3 × 3 × 3 mm piece of in vivo expanded PDX was orthotopically engrafted into the mammary fat pad of 6-to-8-week-old female NSG mice. When tumors developed, the mice were divided into groups using a stratified randomization strategy (*n* ≥ 6 mice/group) and treated as indicated. Tumor volumes were measured using a digital caliper and calculated as described^34^.

In compliance with our institutional animal care and use committee (IACUC) regulations, when a tumor reached >2 cm in diameter or a maximal total photon nr/sec^2^ of 1 × 10^10^, the mouse or all mice in the same experiment were euthanized with CO_2_ euthanasia and the death was confirmed by cervical dislocation.

### In vivo stressing of tumors by intratumoral delivery of DSF/Cu and IR

Treated tumors were intratumorally injected (i.t.) at multiple sites with 200 µL DSF/Cu (1.5 µM/1 µM) or vehicles (DMSO/PBS), followed by 12 Gy local IR on the next day.

### Systemic CAR T therapy

Freshly thawed and expanded (within 2–3 days) CAR T cells were infused i.v. into the mouse tail vein once at the indicated dose.

### In vivo evaluation of JAK inhibitor on stressed tumor-reprogrammed CAR T/TME

The SUM159 mouse tumor model was established and divided into groups (*n* = 5 mice/group) as described above. DSF/Cu (1.5 µM/1 µM) was intratumorally injected on day 14, followed by 12 Gy IR in the tumor the next day. On day 16, 5 × 10^6^ B7-H3 CAR T cells/mouse were infused via the tail vein. The JAKi ruxolitinib (60 mg/kg) was given intraperitoneally daily for 5 days in two schedules as follows: the initiation of the JAKi administration 1 day (termed as JAKi 1) or 5 days (termed as JAKi 5) after CAR T cell infusion^51^.

### Detecting phenotypes and expansion of CAR T in vivo

Fifty microliters of peripheral blood was collected once a week from each mouse, after which red blood cells were removed using ACK lysing buffer (Themo Fisher). CAR T cells were assessed with CD3 and CD45 antibodies in blood and tissues from NSG mice and CD3 and CD45 antibodies and FITC-human B7-H3 (4Ig) / B7-H3b protein in tissues from humanized mice or CD3, CD45, CD62L, CD45RA, PD-1, LAG-3, and TIM3 antibodies and quantified using CountBright Absolute Counting Beads (Thermo Fisher) on a BD LSRII flow cytometer.

### Quantification of cytokines and chemokines

Human cytokines and chemokines found in the cell culture supernatants (undiluted) and tumor tissue homogenates were quantified using a customized 25-plex Luminex panel (Thermo Fisher Scientific) containing the following analytes: CCL11, GM-CSF, Granzyme B, HSP60, IFNα, IFNγ, IL-1, IL-3, IL-4, IL-6, IL-7, IL-8 (CXCL8), IL-9, IL-10, IL-12p70, IL-15, IL-17A, IL-18, CXCL-10, M-CSF, CXCL9, CCL3, CCL4, CCL5, TNFα. The chemokines/cytokines quantification data were collected using MAGPIX xPONENT 4.2 System (VERSION 4.2.1324.0). Data were analyzed using the ProcartaPlex analysis app (VERSION 1.0).

### Immunohistochemistry

For immunohistochemistry (IHC) staining, 4 µm OCT-embedded frozen sections were stained using VECTASTAIN ABC Kits (Vector Laboratories) according to the manufacturer’s instructions. Briefly, slides were washed with 0.5% BSA four times between each step. Slides were blocked with diluted blocking serum for 1 h at room temperature, followed by incubation with 376.96 (1 µg/mL) or CD3 (Biolegend, clone: OKT3, 1:100) antibodies diluted in 2.5% normal horse serum in a humid chamber at 4 °C overnight. The slides were then incubated with biotinylated secondary antibody in 2.5% normal horse serum and subsequently incubated with VECTASTAIN ABC Reagent for 30 min at room temperature. Slides were developed with DAB substrate (Dako) and counterstained with Hematoxylin (Sigma).

### Statistics

Statistical analysis was performed using GraphPad Prism 8 software (VERSION 8.0.2, GraphPad Software Inc.). The one-way ANOVA was used to determine the comparisons among three or more groups at a certain condition. The student’s *t*-test was applied to the comparison between two groups. Additional two-way ANOVA was adopted as specified. Overall survival was calculated using Kaplan–Meier methods and log-rank tests. All data are expressed as mean ± standard deviation (SD) unless specified otherwise. Differences between groups were considered significant when *p* < 0.05.

### Reporting summary

Further information on research design is available in the Nature Portfolio Reporting Summary linked to this article.

### Supplementary information

Supplementary Information

Description of Additional Supplementary Files

Supplementary Data 1

Reporting Summary

### Source data

Source Data

## Data Availability

The raw RNA-seq sequence data generated in this study have been deposited in the Genome Sequence Archive^52^ in National Genomics Data Center^53^, under the accession numbers HRA004874 (for target cancer cells) and HRA004878 (for CAR T cells). The patient information are listed in Supplementary Table 1 and Supplementary Data 1. The remaining data are available within the Article, Supplementary information, and Source Data file. Source data are provided with this paper.
