# Supplementary material for: Stressed target cancer cells drive nongenetic reprogramming of CAR T cells and solid tumor microenvironment
Source: Nat Commun. 2023 Sep 15;14:5727. doi: 10.1038/s41467-023-41282-x (PMC10504259; doi:10.1038/s41467-023-41282-x)
Supplement: Supplementary file 1 — Supplementary Information [file 41467_2023_41282_MOESM1_ESM.pdf]

Supplementary information

Stressed target cancer cells drive nongenetic reprogramming of CAR T cells and solid tumor microenvironment

Yufeng Wang[1,2], David L. Drum[1], Ruochuan Sun[1,3], Yida Zhang[1], Feng Chen[1], Fengfei Sun[1], Emre Dal[1], Ling Yu[1], Jingyu Jia[1], Shahrzad Arya[1], Lin Jia[1], Song Fan[1], Steven J Isakoff[4], Allison M Kehlmann[4], Gianpietro Dotti[5], Fubao Liu[6], Hui Zheng[7], Cristina R Ferrone[8], Alphonse G Taghian[9], Albert B DeLeo[1], Marco Ventin[1], Giulia Cattaneo[1], Yongxiang Li[3], Youssef Jounaidi[10], Peigen Huang[9], Cristina Maccalli[11] Hanyu Zhang[1], Cheng Wang[12], Jibing Yang[13], Genevieve M Boland[1], Ruslan I Sadreyev[14], LaiPing Wong[14], Soldano Ferrone[1,15], Xinhui Wang[1]*

1. Division of Gastrointestinal and Oncologic Surgery, Department of Surgery, Massachusetts General Hospital, Harvard Medical School, Boston, MA, United States
2. Department of General Surgery, Tongji Hospital, School of Medicine, Tongji University, Shanghai, China
3. Department of Gastrointestinal Surgery and General Surgery, First Affiliated Hospital of Anhui Medical University, Hefei, Anhui, China
4. Termeer Center for Targeted Therapies, Massachusetts General Hospital Cancer Center, Boston, MA, United States
5. Lineberger Comprehensive Cancer Center and Department of Microbiology and Immunology, University of North Carolina, Chapel Hill, NC, United States
6. Department of Hepatobiliary & Pancreatic Surgery and Liver Transplantation, Anhui Medical University, Anhui, China
7. Biostatistics Center, Massachusetts General Hospital, Harvard Medical School, Boston, MA, United States
8. Department of Surgery, Cedars - Sinai Medical Center, Los Angeles, CA, United States
9. Department of Radiation Oncology, Massachusetts General Hospital, Harvard Medical School, Boston, MA, United States
10. Department of Anesthesia, Critical Care and Pain Medicine, Massachusetts General Hospital, Harvard Medical School, Boston, MA, United States
11. Research Department, Sidra Medicine, Doha, Qatar
12. Vincent Center for Reproductive Biology, Vincent Department of Obstetrics and Gynecology, Massachusetts General Hospital, Harvard Medical School, Boston, MA, United States
13. Center for Comparative Medicine, Massachusetts General Hospital, Harvard Medical School, Boston, MA, United States
14. Department of Molecular Biology, Massachusetts General Hospital, Harvard Medical School, Boston, MA, United States
15. Department of Orthopaedics, Massachusetts General Hospital, Boston, MA, United States

*** Correspondence:**
Xinhui Wang**;** xwang30@mgh.harvard.edu

Supplementary information includes:

Supplementary Figures 1 to 12 and Supplementary Table 1 to 2

Supplementary Figure 1

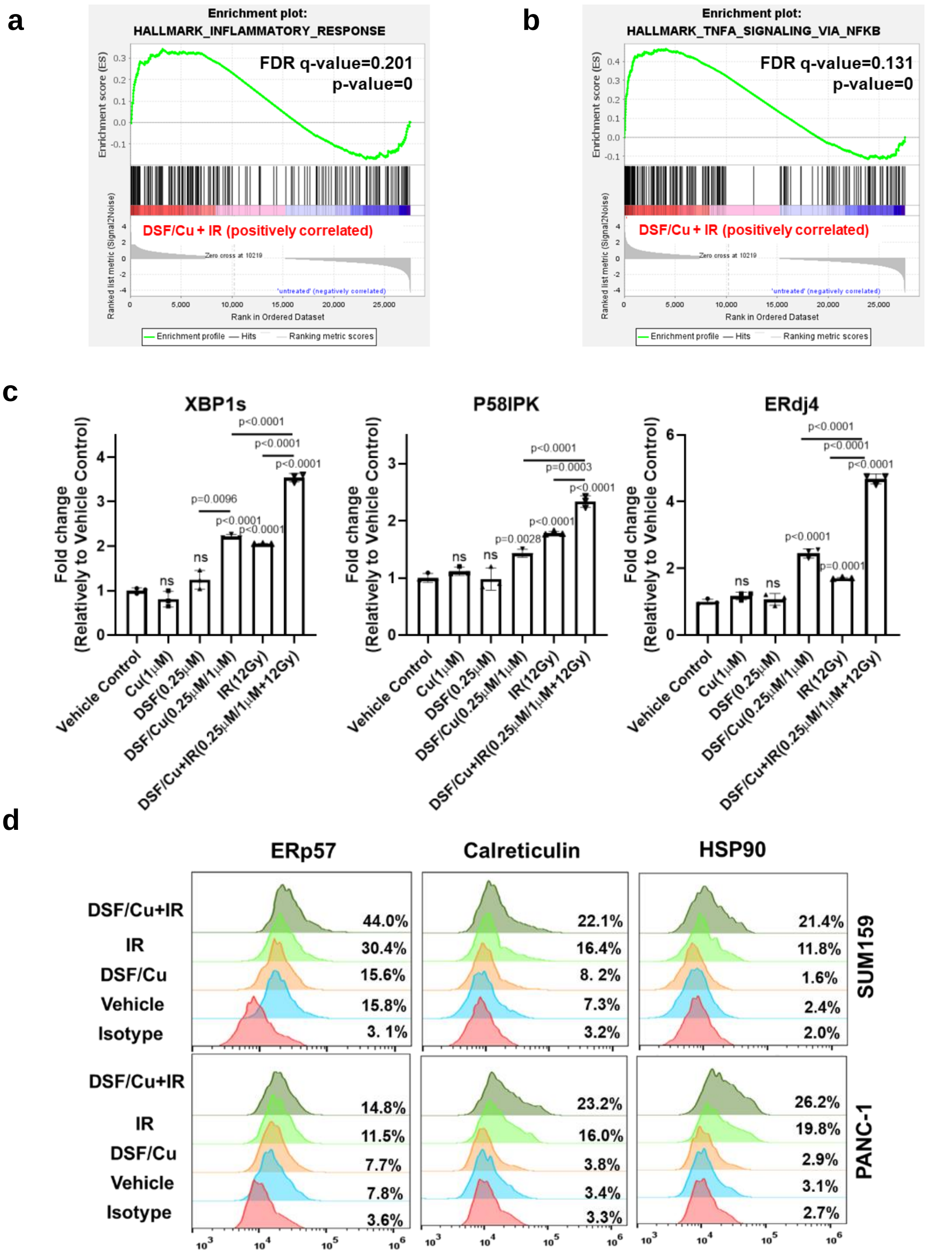

Supplementary Fig 1. DSF/Cu+IR induces cellular stress responses in target cancer cells *in vitro*. Representative gene set enrichment analysis (GSEA) plots illustrating **a,** "INFLAMMATORY_RESPONSE" (n=3 biologically independent experiments, GSEA-computed p values and false discovery rate). **b,** "TNFA_SIGNALING_VIA_NFKB" in DSF/Cu and IR-stressed SUM159 tumor cells. (n=3 biologically independent experiments, GSEA-computed p values and false discovery rate). **c,** Detection by qRT-PCR of DSF/Cu+IR-induced ER stress indicator XBP1s mRNA and its downstream target genes ERdj4, P58IPK in PANC-1 cells (n=3 independent experiments), and **d,** The expression level of stress-related markers ERp57, calreticulin, and HSP90 were measured using flow cytometry after indicated treatments (n=3 independent experiments). Statistical comparisons were performed using one-way ANOVA with Tukey's multiple comparisons test (**c**). P values are shown and error bars indicate mean ± SD. Source data are provided as a Source Data file.

Supplementary Figure 2

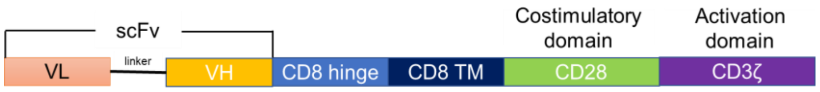

Supplementary Fig 2. The schema of the CAR construct. B7-H3 -specific single chain of variable region (scFv) 376.96[53], CSPG4-specific scFv 763.74[54] and CD19-specific scFv MFC63[55] were used for each CAR construction as described[53]. The amino acid sequences of the CAR constructs are available for B7-H3 (US10519214B2), CSPG4 (US20210252067A1) and CD19 (US9701758B2).

Supplementary Figure 3

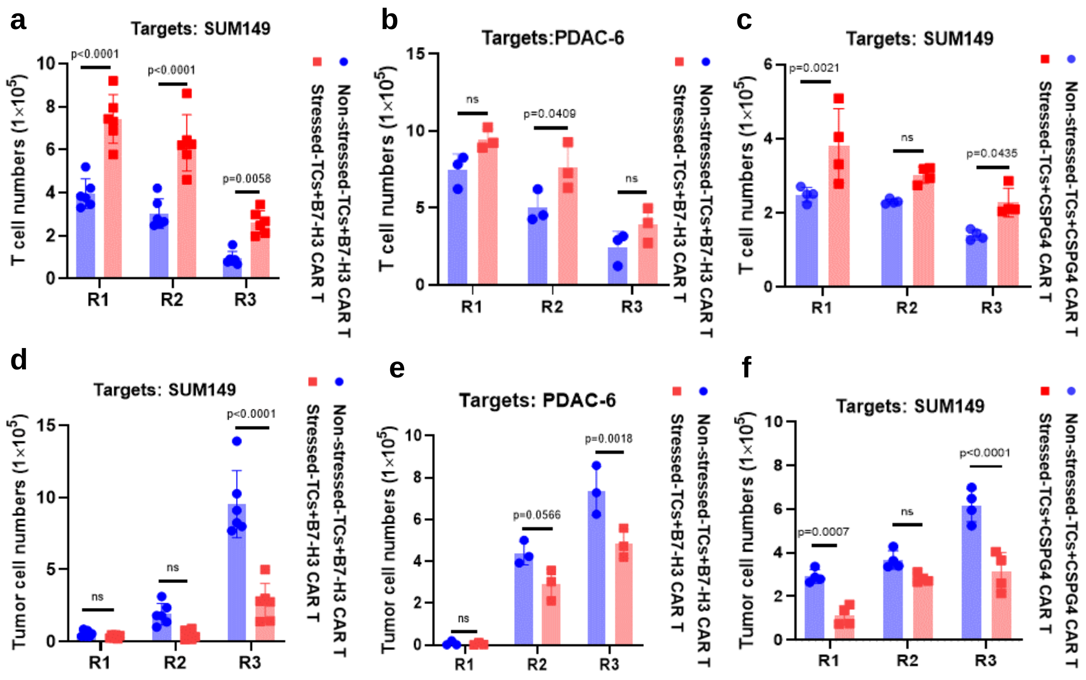

Supplementary Fig 3. DSF/Cu+IR-stressed target cells promote functional switch in CAR T cells with profoundly enhanced *in vitro* expansion and cytotoxicity. The absolute number of CAR T cells and target tumor cells was counted after each round of repetitive co-culture assay (E:T=1:2) in the non-stressed tumor cells and CAR T group and in the DSF/Cu+IR-treated stressed tumor cells and CAR T group. **a, d,** Target cells: SUM149, effector cells: B7-H3 CAR T cells (n=6 independent experiments); **b, e,** Target cells: PDAC-6, effector cells: B7-H3 CAR T cells (n=3 independent experiments); **c, f,** Target cells: SUM149, effector cells: CSPG4 CAR T cells (n=4 independent experiments). Statistical comparisons were performed using two-way ANOVA with Sidak's multiple comparisons test (**a**, **b**, **c**, **d**, **e**, **f**). P values are shown and error bars indicate mean ± SD. ns represents no significant difference. Source data are provided as a Source Data file.

Supplementary Figure 4

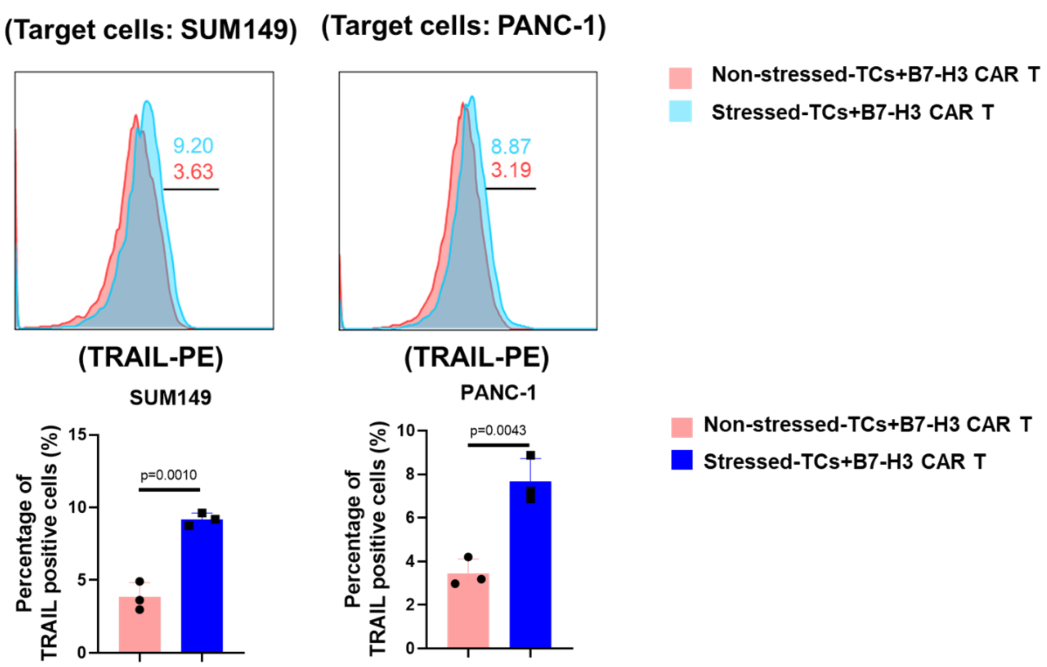

Supplementary Fig 4. DSF/Cu+IR-stressed target cells promote TRAIL expression on their co-cultured CAR T cells. The percentage of TRAIL expressed on B7-H3 CAR T cells after 3 days of co-culture with different DSF/Cu+IR-stressed target cells (SUM149 and PANC-1, n=3 independent experiments). Statistical comparisons were performed using two-tailed unpaired t test. P values are shown and error bars indicate mean ± SD. Source data are provided as a Source Data file.

Supplementary Figure 5

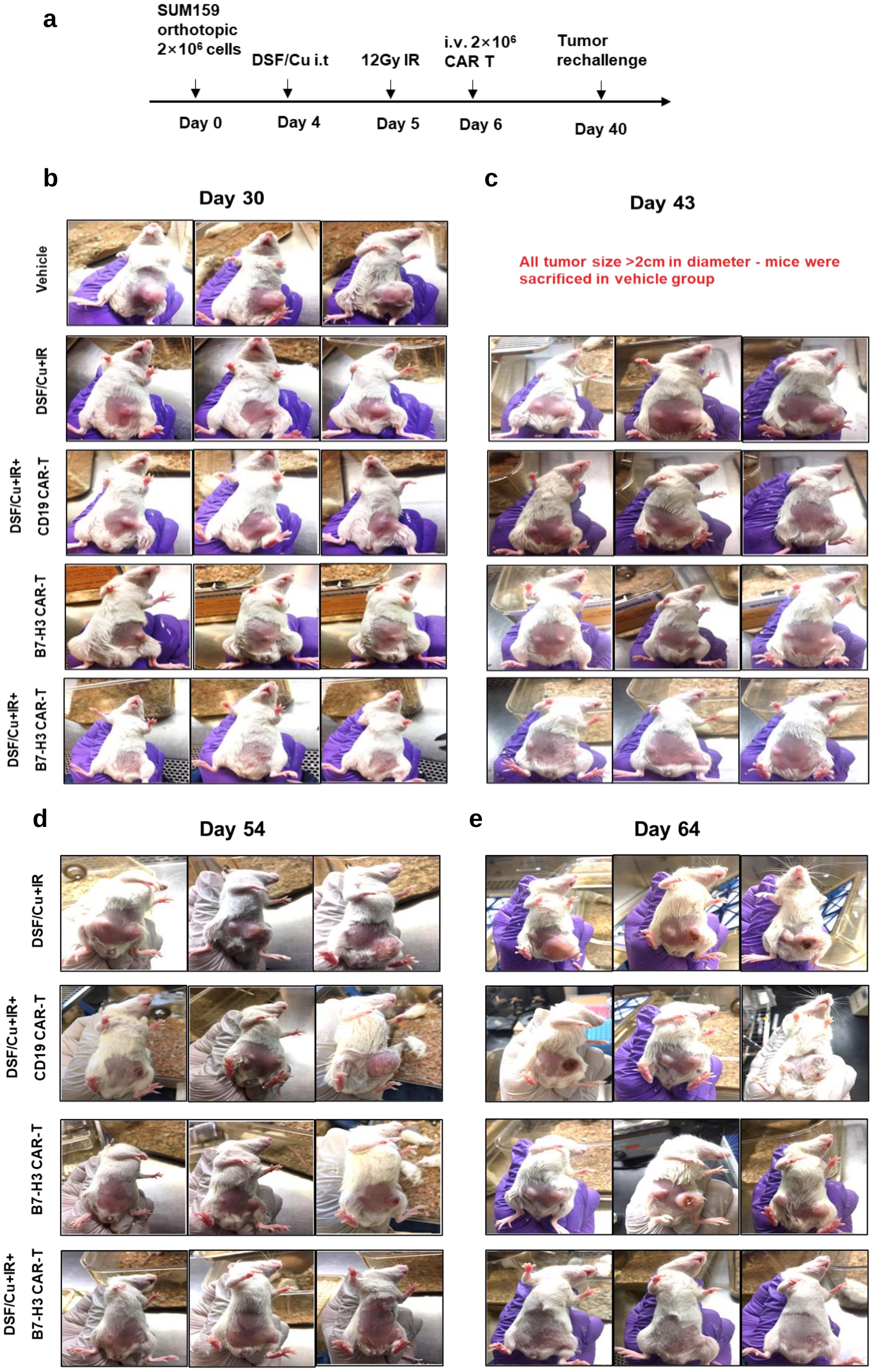

Supplementary Fig 5. Long-term primary tumor regression and time-dependent rechallenged tumor rejections in response to the DSF/Cu+IR +CAR T therapy reflect the expansion of *in vivo* persisted early memory CAR T cells. a, Schema of the TNBC orthotopic xenograft model (SUM159) with indicated treatments. Representative images of **b,** complete primary tumor rejection in 100% mice treated with DSF/Cu+IR+CAR T on day 30 (right side), **c,** 100% tumor formation in mice after SUM159 cell-rechallenge on day 43 (left side), **d,** rejection of 40% rechallenged tumors in mice treated with DSF/Cu+IR+CAR T on day 54 and **e,** rejection of 100% rechallenged tumors in mice treated with DSF/Cu+IR+CAR T on day 64 (n=5 mice/group).

Supplementary Figure 6

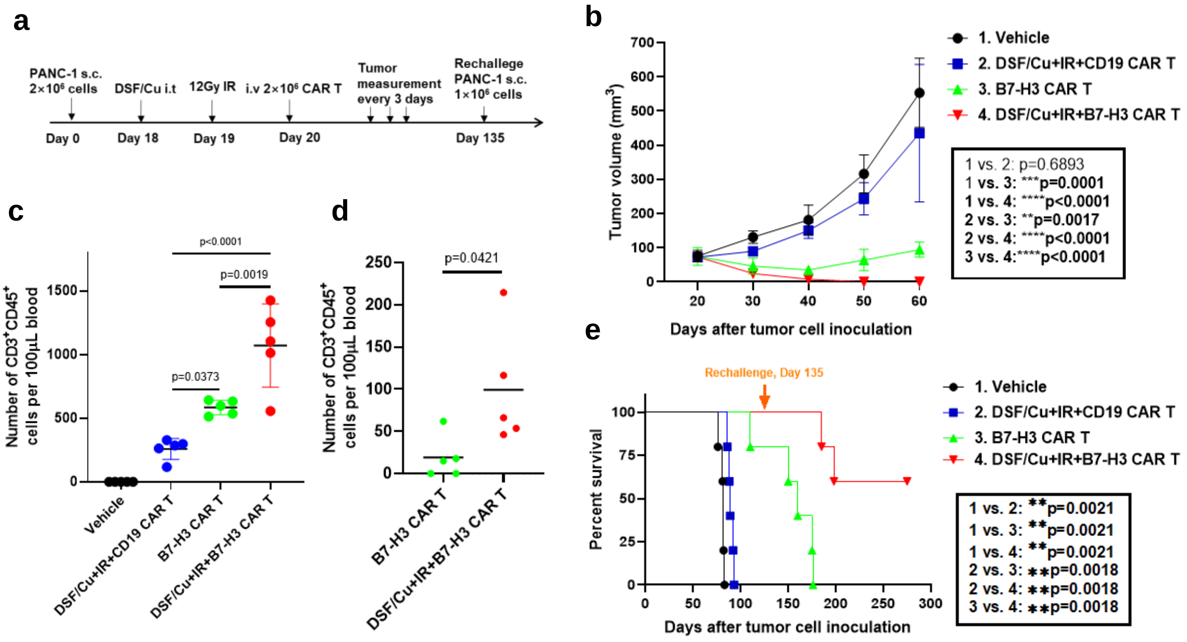

Supplementary Fig 6. CAR T cells reprogrammed *in vivo* via target cancer cells stressed by intratumoral delivery of DSF/Cu and tumor localized IR induce potent, sustained and memory anti-solid tumor responses in PDAC xenograft mouse models. a, Schematic representation of PDAC xenograft model (PANC-1) infused with CAR T cells on day 20 after tumor cell inoculation. **b,** Tumor volumes (n=5 mice/group) in the PDAC xenograft tumor model. **c, d,** Frequency of human $CD3^{+}CD45^{+}$ CAR T cells in blood collected on days 8 (**c**) and 92 (**d**) after CAR T cell primary inoculation (n=5 mice/group). **e,** Kaplan-Meier survival curve of mice after tumor rechallenge (n=5 mice/group). Statistical comparisons were performed using two-way ANOVA with Tukey's multiple comparisons test (**b**), one-way ANOVA with Tukey's multiple comparisons test (**c**), two-tailed unpaired t test (**d**) and log-rank test (**e**). P values are shown and error bars indicate mean ± SD. Source data are provided as a Source Data file.

Supplementary Figure 7

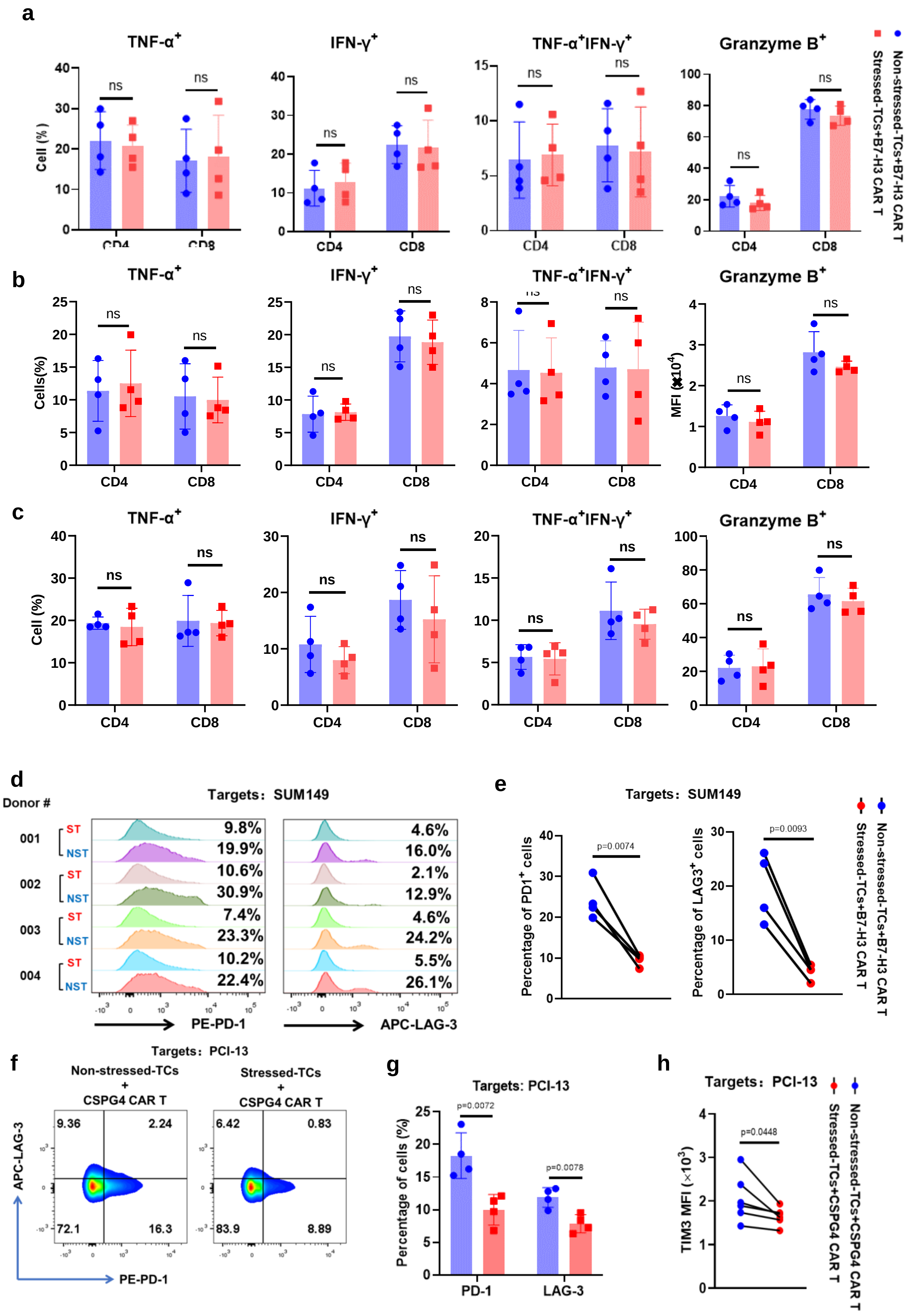

Supplementary Fig 7. DSF/Cu+IR-stressed target cells promote functional switch in CAR T cells with less *in vitro* exhaustion, defined by markers associated with T cell exhaustion. a, b, c, B7-H3 CAR T cells were intracellularly stained for TNF-α, IFN-γ, and Granzyme B after the 24h co-culture experiment (Target cells: **a,** SUM149, **b,** SUM159, **c,** PANC-1) (n=4 independent experiments). **d, e** Exhausted CAR T cells (%), defined as $CD3^{+}PD\text{-}1^{+}$ or $CD3^{+}LAG\text{-}3^{+}$ after co-cultured with non-stressed SUM149 tumor cells (NST) and stressed SUM149 tumor cells (ST) at the end of round 3 of the repetitive co-culture assay (n=4 different PBMC donors from independent experiments). **f, g, h,** Exhausted CAR T cells (%), defined as $CD3^{+}PD\text{-}1^{+}$ or $CD3^{+}LAG\text{-}3^{+}$ (**f, g,** n=4), and $CD3^{+}$ $TIM3^{+}$ (**h**) after co-cultured with non-stressed cells and stressed target cells at the end of round 3 of the repetitive co-culture assay (n=4 independent experiments). Statistical comparisons were performed using two-tailed unpaired t test (**a**, **b**, **c**, **g**), two-tailed paired t test (**e**, **h**). P values are shown and error bars indicate mean ± SD. ns represents no significant difference. Source data are provided as a Source Data file.

Supplementary Figure 8

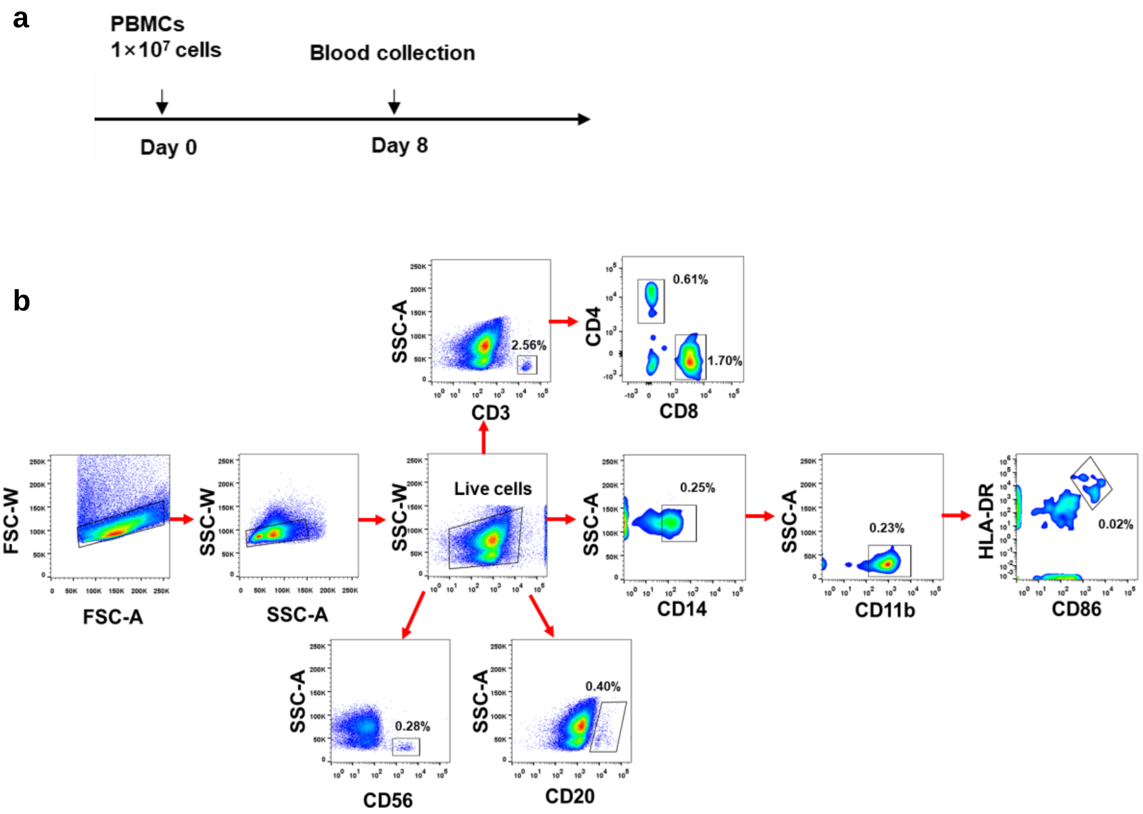

Supplementary Fig 8. Mouse humanization with human PBMCs. a. Schema of mouse humanization. **b,** various types of engrafted human immune cells were detected in mouse peripheral blood 8 days post-humanization. Gating strategy: cells were gated on forward (FSC-A) and side (SSC-A) scatter, followed by gating on single cells (FSC-A, FSC-W and SSC-A, SSC-W) and live cell population based on Viability Dyes staining. Live cells were gated on anti-human CD3, CD4, CD8 for T cell population; anti-human CD56 for natural killing cells; anti-human CD20 for B cells; anti-human CD14 for monocytes and anti-human CD14, CD11b, CD86, HLA-DR for dendritic cells (DCs).

Supplementary Figure 9

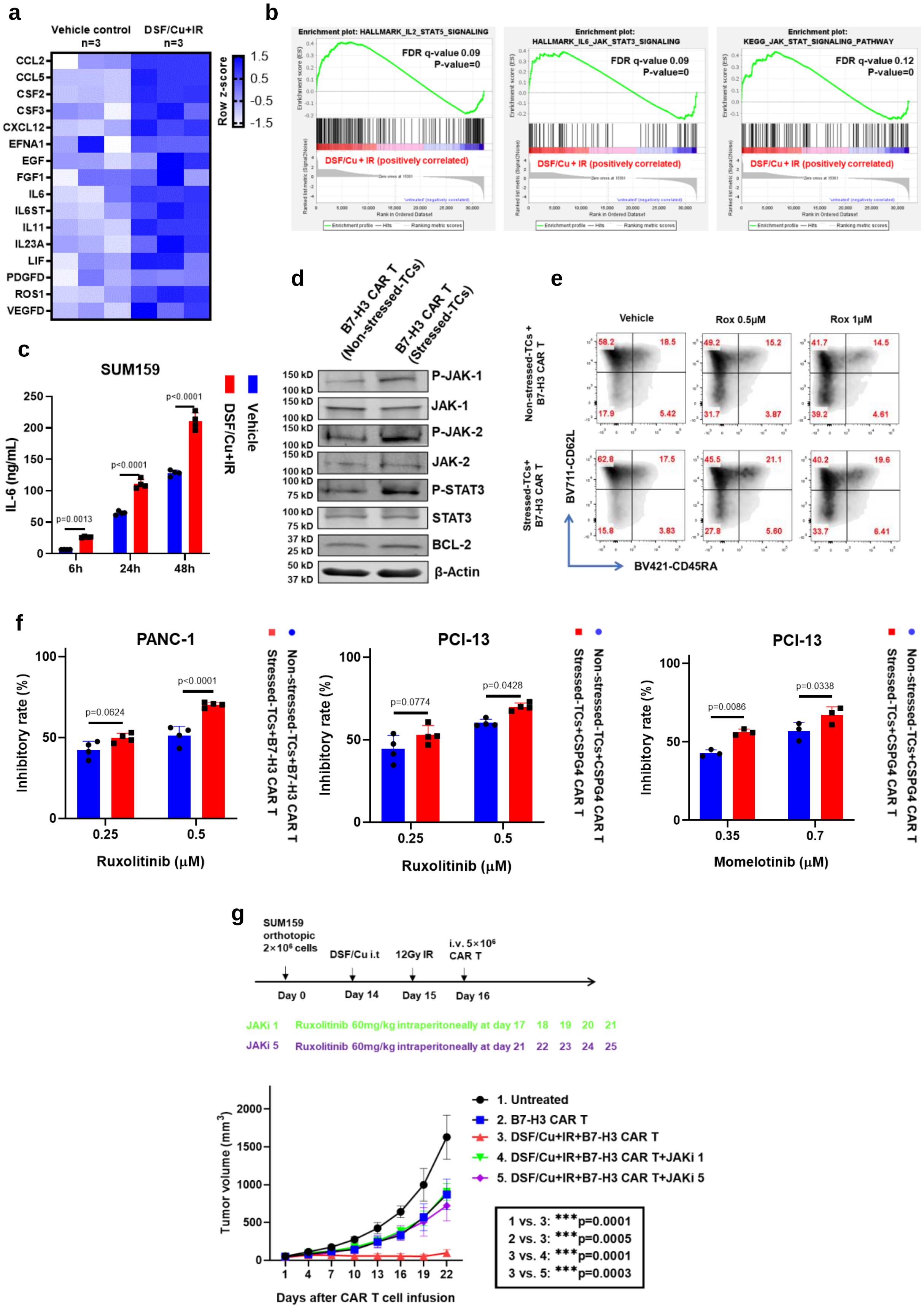

Supplementary Fig 9. The JAK/STAT signaling axis is implicated in stressed cancer cell-induced phenotypic and functional switches of CAR T cells. a, RNA-seq analysis of genes leading to activation of JAK/STAT pathways in DSF/Cu+IR stressed-SUM159 tumor cells (n=3 biologically independent experiments). **b,** Representative GSEA enrichment plot illustrates the JAK/STAT signaling pathway in RP- vs. NRP-B7-H3 CAR T cells (n=3 biologically independent experiments, GSEA-computed p values and false discovery rate). **c**. Increased and time-dependent IL-6 release in the supernatant of SUM159 cells after stressed by DSF/Cu+IR treatment measured by ELISA. (n=3 independent experiments) **d,** Activation of JAK/STAT pathway in B7-H3 CAR T cells after 48h of co-cultured with stressed target cells SUM159 (n=3 independent experiments) **e,** JAK/STAT inhibitor ruxolitinib decreased $CD62L^{+}CD45RA^{-}$ T_{CM} (%) in RP- and NRP- B7-H3 CAR T cells (n=3 independent experiments) **f,** The inhibitory rates of killing target cells by B7-H3 CAR T cells (E:T=1:2) in the presence or absence of JAK/STAT inhibitors ruxolitinib (n=4 independent experiments with PANC-1 and PCI-13 cell lines) or momelotinib (n=3 independent experiments with PCI-13 cell line). **g**, JAK/STAT inhibitor ruxolitinib used at both schedules, i.e., starting day 1 or 5 post-CAR T cell infusion completely abolished the anti-tumor effect mediated by stressed tumor-reprogrammed CAR T/TME (n=5 mice/group). Data were collected from at least 3-4 independent experiments. Statistical comparisons were performed using two-way ANOVA with Sidak's multiple comparisons test (**c**, **f**), two-way ANOVA with Tukey's multiple comparisons test (**g**). P values are shown and error bars indicate mean ± SD. Source data are provided as a Source Data file.

Supplementary Figure 10

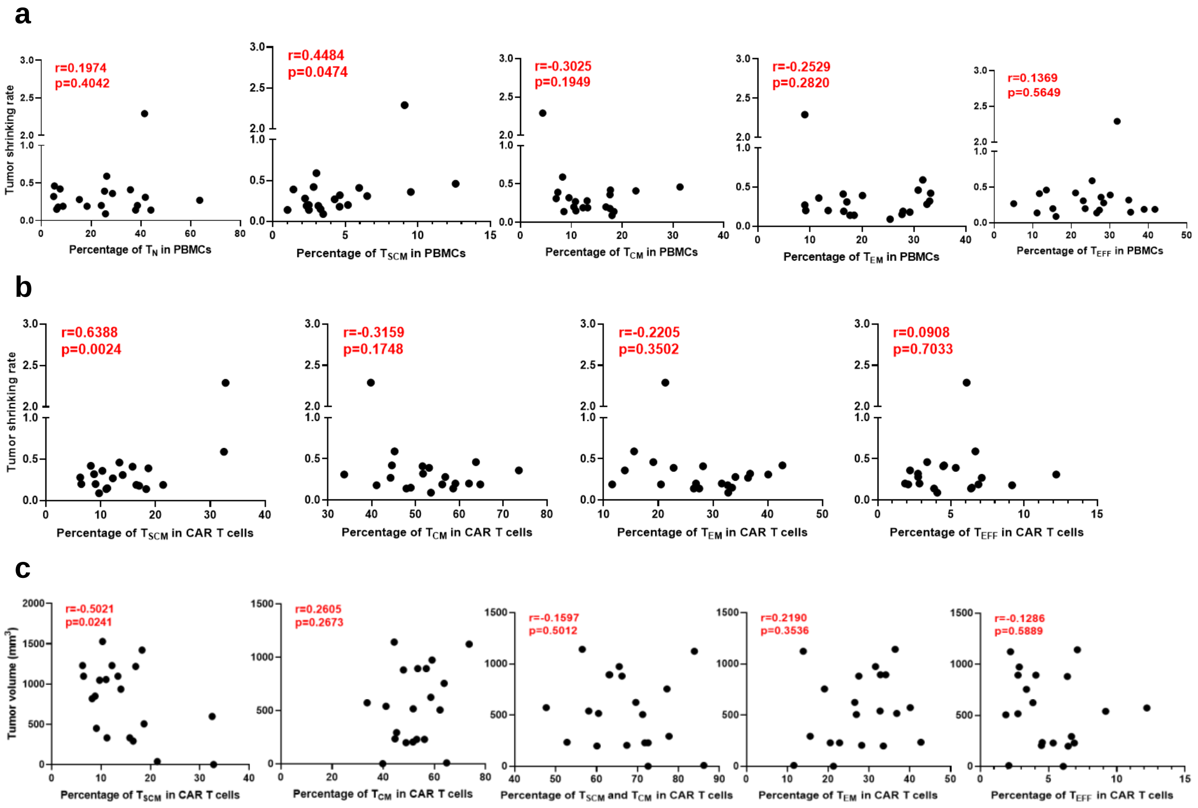

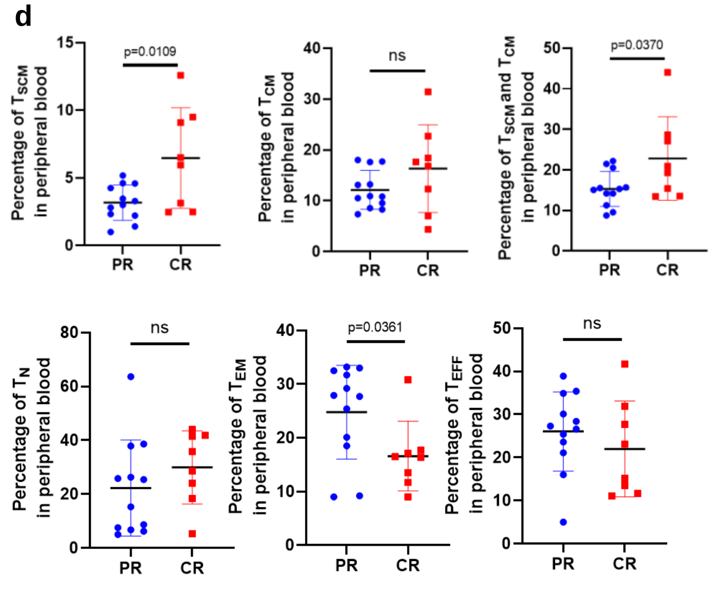

e

Patient #	B7-H3 CAR T Tumor volume (mm^3, day 28)	DSF/Cu+IR+B7-H3 CAR T Tumor volume (mm^3, day 28)
1	0	0
2	292.5	0
3	598	219.375
4	450.84	0
5	332.8	263.25
6	1421.55	0
7	849.42	121.68
8	37.44	71.5
9	1528.8	0
10	1096.875	0
11	936	0
12	1056.25	421.2
13	332.8	0
14	1230.32	292.5
15	1096.875	263.25
16	1230.32	178.36
17	1048.32	203.84
18	817.96	488.41
19	505.44	421.2
20	1218.75	37.44

Supplementary Fig 10. **T cell subtypes are closely correlated with therapeutic responses against solid tumors by RP B7-H3 CAR T derived from PBMCs of patients with metastatic breast cancer. a,** The Pearson r correlation (two-tailed) between the percentage of T_{SCM} / T_{CM} / T_{EM} / T_{EFF} in PBMCs and the anti-tumor response *in vivo*, calculated using the equation: tumor shrinking rate = (tumor volume on day 4) / (tumor volume on day 12) (n=20 mice). **b, c** The Pearson r correlation (two-tailed) between the percentage of T_{SCM} ($CD3^+CD62L^+CD45RA^+$) / T_{CM} ($CD3^+CD62L^+CD45RA^-$) / T_{EM} ($CD3^+CD62L^-CD45RA^-$)/ T_{EFF} ($CD3^+CD62L^-CD45RA^+$) in CAR T cells and the anti-tumor response *in vivo* (n=20 mice). **d,** The percentage of total T cells (T_N /T_{SCM} / T_{CM} / T_{EM} / T_{EFF}) in the peripheral blood of CR (n=8 mice) as compared to PR (n=12 mice). **e,** Tumor volumes from individual mice were measured on day 28 post CAR T cell injection. CR: complete responders. PR: partial responders. Statistical comparisons were performed using two-tailed Pearson r correlation test (**a**, **b**, **c**), two-tailed unpaired t test (**d**). P values are shown and error bars indicate mean ± SD. Source data are provided as a Source Data file.

Supplementary Figure 11

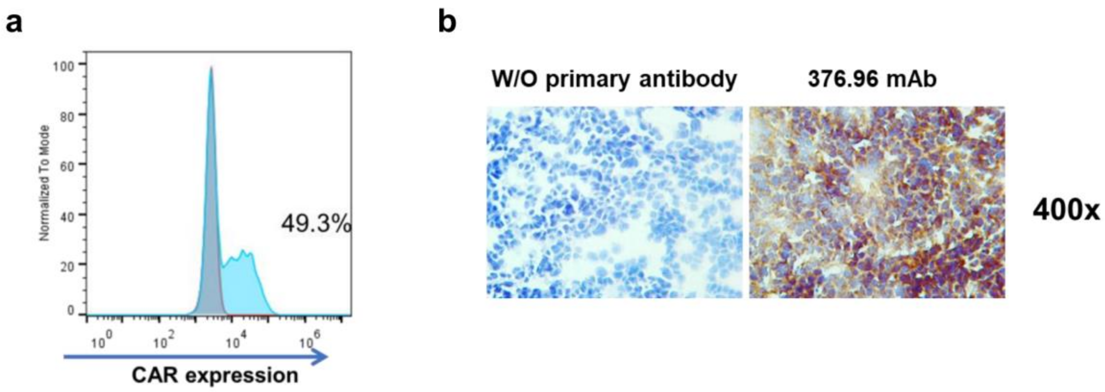

Supplementary Fig 11. Patient-derived B7-H3 CAR T cells and PDX tissue. **a,** Transduction efficiency of B7-H3 CAR T cells derived from PBMCs of the metastatic breast cancer patient. These CAR T cells were used for the experiment outlined in Fig6j-m. **b,** B7-H3 expression was detected in the frozen PDX tissue (TNBC, the PDX mouse model used in Fig6j-m) by immunohistochemical staining with B7-H3-specific mAb 376.96 (2µg/mL).

Supplementary Figure 12

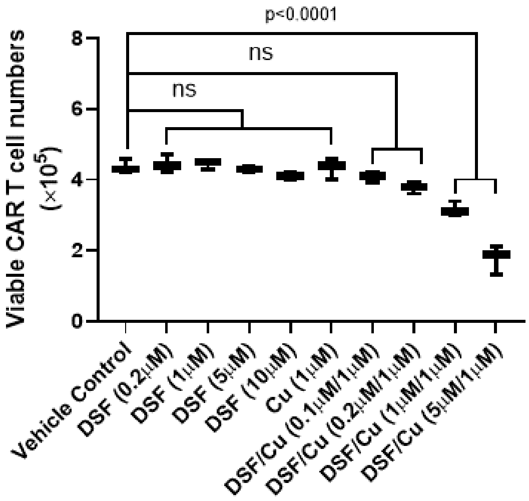

Supplementary Fig 12. The effect on cell viability of DSF/Cu vs. DSF on B7-H3 CAR T cells. B7-H3 CAR T (2×10^6 cells/well) were seeded in a 6-well plate and incubated with vehicle (DMSO) or DSF or Cu or DSF/Cu at indicated concentrations for 2h at 37°C in 5% CO_2. The total numbers of live cells were counted using Trypan blue exclusion method (n=3 independent experiments). Statistical comparisons were performed using one-way ANOVA with Tukey's multiple comparisons test. P values are shown and error bars indicate mean ± SD. ns represents no significant difference. Source data are provided as a Source Data file.

Supplementary Table 1, TSCM (%) in each patient

ID	Metastasis	Age	ER	PR	HER2	Invasive histology (Ductal/Lobular/Mixed/NOS)	Therapy (at time of blood collection)	T_{SCM}(%)
1	Yes	62	+	+	+	Ductal	Trastuzumab/Pertuzumab/Docetaxel	9.04
2	Yes	75	+	+	+	Ductal	Ipatasertib/Palbociclib/Fulvestrant	8.32
3	Yes	68	+	-	+	Lobular	H3B-6545	4.82
4	Yes	43	+	+	+	Ductal	Trastuzumab	5.61
5	No	33	+	+	+	Ductal	Trastuzumab/Paclitaxel/Pertuzumab	3.52
6	Yes	58	+	+	+	Ductal	Ribociclib/Exemestane	3.45
7	Yes	59	+	+	-	Ductal	Exemestane/Everolimus/ LEE011	3.30
8	No	64	+	+	+	Ductal	Trastuzumab	3.28
9	Yes	72	+	+	+	Lobular	JTX-2011/Nivolumab	2.86
10	No	34	-	+	+	Ductal	Trastuzumab	2.47
11	Yes	66	+	+	-	Ductal	Ipatasertib/Arimidex	1.91
12	Yes	57	+	+	+	Ductal	Trastuzumab	1.85
13	Yes	55	+	+	+	Ductal	H3B-6545	1.80
14	No	34	+	+	+	Ductal	Adrimycin/Cytoxan	1.63
15	Yes	72	+	+	+	Lobular	Ipatasertib/Arimidex	1.24
16	Yes	72	+	+	+	Ductal	Ipatasertib/Exemestane	1.07
17	Yes	51	+	+	+	Ductal	GDC0032/Fulvestrant	0.99
18	Yes	58	+	+	+	NOS	GDC-927	0.64
19	Yes	41	+	+	+	Lobular	Trastuzumab/Pertuzumab/Paclitaxel	0.62
20	Yes	60	+	+	-	Ductal	Ipatasertib/Palbociclib/Fulvestrant	0.61

Supplementary Table 2, List of gene sets for heatmaps chosen for The Molecular Signature Database (MSiDB)

Name	Gene sets
ER stress	GOBP_ATF6_MEDIATED_UNFOLDED_PROTEIN_RESPONSE; GOBP_RESPONSE_TO_ENDOPLASMIC_RETICULUM_STRESS;
Oxidative stress	Gene Set CHUANG_OXIDATIVE_STRESS_RESPONSE_UP; GOBP_CELL_DEATH_IN_RESPONSE_TO_OXIDATIVE_STRESS; Oxidative Stress Induced Gene Expression Via Nrf2
Chemical stress	REACTOME_CELLULAR_RESPONSE_TO_CHEMICAL_STRESS
Heat shock stress	REACTOME_CELLULAR_RESPONSE_TO_HEAT_STRESS
CAR-T proliferation	GOBP_ACTIVATED_T_CELL_PROLIFERATION; GOBP_B_CELL_PROLIFERATION_INVOLVED_IN_IMMUNE_RESPONSE; GOBP_IMMATURE_T_CELL_PROLIFERATION; GOBP_POSITIVE_REGULATION_OF_ACTIVATED_T_CELL_PROLIFERATION; GOBP_T_CELL_PROLIFERATION_INVOLVED_IN_IMMUNE_RESPONSE
CAR-T activation/effector function	GO_POSITIVE_REGULATION_OF_LYMPHOCYTE_ACTIVATION; GOBP_T_CELL_ACTIVATION_INVOLVED_IN_IMMUNE_RESPONSE; GOBP_T_CELL_ACTIVATION_VIA_T_CELL_RECEPTOR_CONTACT_WITH_ANTIGEN_BOUND_TO_MHC_MOLECULE_ON_ANTIGEN_PRESENTING_CELL
CAR-T exhaustion	GSE9650_EXHAUSTED_VS_MEMORY_CD8_TCELL_UP; GSE9650_EFFECTOR_VS_EXHAUSTED_CD8_TCELL_DN; GSE41867_MEMORY_VS_EXHAUSTED_CD8_TCELL_DAY30_LCMV_UP
CAR-T memory	GSE3982_CENT_MEMORY_CD4_TCELL_VS_TH2_UP; GSE3982_EFF_MEMORY_VS_CENT_MEMORY_CD4_TCELL_UP; GSE9650_EFFECTOR_VS_MEMORY_CD8_TCELL_UP; GSE10239_MEMORY_VS_KLRG1HIGH_EFF_CD8_TCELL_UP; GSE23321_CD8_STEM_CELL_MEMORY_VS_CENTRAL_MEMORY_CD8_TCELL_UP; GSE23321_CD8_STEM_CELL_MEMORY_VS_EFFECTOR_MEMORY_CD8_TCELL_UP
