# Supplementary material for: Stressed target cancer cells drive nongenetic reprogramming of CAR T cells and solid tumor microenvironment
Source: Nat Commun. 2023 Sep 15;14:5727. doi: 10.1038/s41467-023-41282-x (PMC10504259; doi:10.1038/s41467-023-41282-x)
Supplement: Supplementary file 2 — Description of Additional Supplementary Files [file 41467_2023_41282_MOESM2_ESM.pdf]

Description of Additional Supplementary Files

Title: **Supplementary Data 1**, Patient demographics and characteristics

Description: This table includes 21 patients with metastatic breast cancer, most of whom were heavily pretreated. All of them were used to generate CAR T cells for *in vivo* experiments.
