# Supplementary material for: Stressed target cancer cells drive nongenetic reprogramming of CAR T cells and solid tumor microenvironment
Source: Nat Commun. 2023 Sep 15;14:5727. doi: 10.1038/s41467-023-41282-x (PMC10504259; doi:10.1038/s41467-023-41282-x)
Supplement: Supplementary file 4 — Reporting Summary [file 41467_2023_41282_MOESM4_ESM.pdf]

nature portfolio

Corresponding author(s): Xinhui Wang

Last updated by author(s): Aug 1, 2023

# Reporting Summary

Nature Portfolio wishes to improve the reproducibility of the work that we publish. This form provides structure for consistency and transparency in reporting. For further information on Nature Portfolio policies, see our Editorial Policies and the Editorial Policy Checklist.

## Statistics

For all statistical analyses, confirm that the following items are present in the figure legend, table legend, main text, or Methods section.

| n/a | Confirmed | |
|---|---|---|
| ☐ | ☒ | The exact sample size (*n*) for each experimental group/condition, given as a discrete number and unit of measurement |
| ☐ | ☒ | A statement on whether measurements were taken from distinct samples or whether the same sample was measured repeatedly |
| ☐ | ☒ | The statistical test(s) used AND whether they are one- or two-sided<br>*Only common tests should be described solely by name; describe more complex techniques in the Methods section.* |
| ☒ | ☐ | A description of all covariates tested |
| ☒ | ☐ | A description of any assumptions or corrections, such as tests of normality and adjustment for multiple comparisons |
| ☐ | ☒ | A full description of the statistical parameters including central tendency (e.g. means) or other basic estimates (e.g. regression coefficient) AND variation (e.g. standard deviation) or associated estimates of uncertainty (e.g. confidence intervals) |
| ☐ | ☒ | For null hypothesis testing, the test statistic (e.g. $F$, $t$, $r$) with confidence intervals, effect sizes, degrees of freedom and $P$ value noted<br>*Give P values as exact values whenever suitable.* |
| ☒ | ☐ | For Bayesian analysis, information on the choice of priors and Markov chain Monte Carlo settings |
| ☒ | ☐ | For hierarchical and complex designs, identification of the appropriate level for tests and full reporting of outcomes |
| ☐ | ☒ | Estimates of effect sizes (e.g. Cohen's *d*, Pearson's *r*), indicating how they were calculated |

*Our web collection on statistics for biologists contains articles on many of the points above.*

## Software and code

Policy information about availability of computer code

| | |
|---|---|
| Data collection | Flow cytometry data were collected using BD FACSDiva software (VERSION 8.0) and BD Accuri C6 software (VERSION 1.0.264.21). Bioluminescence imaging for animal study was collected using Perkin-Elmer IVIS100 imaging system (MA, USA). Western blot data were collected using ODYSSEY Infrared imaging system Application software (VERSION 3.0). The purity and concentration of RNA was estimated using Nanodrop 2000 (Thermo Scientific). The ELISA data were collected using All-In-One Microplate Reader Software Gen5 (VERSION 2.09). The Q-PCR data were collected using LightCycler 96 software (VERSION 1.1). The chemokines/cytokines quantification data were collected using MAGPIX xPONENT 4.2 System (VERSION 4.2.1324.0). |
| Data analysis | FACS data were analyzed by FlowJo software (VERSION 10.8.1, Ashland, OR). Western blot data were analyzed by Image Studio Lite (VERSION 5.2). Statistical analysis was performed using GraphPad Prism 8 software (VERSION 8.0.2, GraphPad Software Inc.) The Q-PCR data were collected using LightCycler 96 software (VERSION 1.1). The quantification of chemokines and cytokines were analyzed using the ProcartaPlex analysis app (VERSION 1.0). The GSEA was performed using the GSEA_4.1.0 software (Broad Institute) on genes from RNA-seq. Bioluminescence imaging from animal studies were analyzed using Aura imaging software (VERSION 3.2). |

For manuscripts utilizing custom algorithms or software that are central to the research but not yet described in published literature, software must be made available to editors and reviewers. We strongly encourage code deposition in a community repository (e.g. GitHub). See the Nature Portfolio guidelines for submitting code & software for further information.

## Data

Policy information about availability of data

All manuscripts must include a data availability statement. This statement should provide the following information, where applicable:

- Accession codes, unique identifiers, or web links for publicly available datasets
- A description of any restrictions on data availability
- For clinical datasets or third party data, please ensure that the statement adheres to our policy

The RNA-sequencing data generated in this study have been deposited in the GSA (Genome Sequence Archive) database under accession codes HRA004874 and HRA004878, which are publicly accessible at https://ngdc.cncb.ac.cn/gsa-human.
List of gene sets for heatmaps chosen from The Molecular Signature Database (MSiDB): GOBP_ATF6_MEDIATED_UNFOLDED_PROTEIN_RESPONSE; GOBP_RESPONSE_TO_ENDOPLASMIC_RETICULUM_STRESS; Gene Set CHUANG_OXIDATIVE_STRESS_RESPONSE_UP; GOBP_CELL_DEATH_IN_RESPONSE_TO_OXIDATIVE_STRESS (GO:0006979); Oxidative Stress Induced Gene Expression Via Nrf2; REACTOME_CELLULAR_RESPONSE_TO_CHEMICAL_STRESS; REACTOME_CELLULAR_RESPONSE_TO_HEAT_STRESS; GOBP_ACTIVATED_T_CELL_PROLIFERATION; GOBP_B_CELL_PROLIFERATION_INVOLVED_IN_IMMUNE_RESPONSE; GOBP_IMMATURE_T_CELL_PROLIFERATION; GOBP_POSITIVE_REGULATION_OF_ACTIVATED_T_CELL_PROLIFERATION; GOBP_T_CELL_PROLIFERATION_INVOLVED_IN_IMMUNE_RESPONSE; GO_POSITIVE_REGULATION_OF_LYMPHOCYTE_ACTIVATION; GOBP_T_CELL_ACTIVATION_INVOLVED_IN_IMMUNE_RESPONSE; GOBP_T_CELL_ACTIVATION_VIA_T_CELL_RECEPTOR_CONTACT_WITH_ANTIGEN_BOUND_TO_MHC_MOLECULE_ON_ANTIGEN_PRESENTING_CELL; GSE9650_EXHAUSTED_VS_MEMORY_CD8_TCELL_UP;
GSE9650_EFFECTOR_VS_EXHAUSTED_CD8_TCELL_DN;
GSE41867_MEMORY_VS_EXHAUSTED_CD8_TCELL_DAY30_LCMV_UP; GSE3982_CENT_MEMORY_CD4_TCELL_VS_TH2_UP;
GSE3982_EFF_MEMORY_VS_CENT_MEMORY_CD4_TCELL_UP;
GSE9650_EFFECTOR_VS_MEMORY_CD8_TCELL_UP;
GSE10239_MEMORY_VS_KLRG1HIGH_EFF_CD8_TCELL_UP;
GSE23321_CD8_STEM_CELL_MEMORY_VS_CENTRAL_MEMORY_CD8_TCELL_UP;
GSE23321_CD8_STEM_CELL_MEMORY_VS_EFFECTOR_MEMORY_CD8_TCELL_UP.
Data availability: The raw RNA-seq sequence data generated in this study have been deposited in the Genome Sequence Archive (Genomics, Proteomics & Bioinformatics 2021) in National Genomics Data Center (Nucleic Acids Res 2022), under the accession numbers HRA004874 (for target cancer cells) and HRA004878 (for CAR-T cells). Both accession codes are publicly accessible at https://ngdc.cncb.ac.cn/gsa-human. The patient information is listed in Supplementary Tables 1 and 2. The remaining data are available within the article, Supplementary information, and source data file. Source data are provided with this paper.

## Research involving human participants, their data, or biological material

Policy information about studies with human participants or human data. See also policy information about sex, gender (identity/presentation), and sexual orientation and race, ethnicity and racism.

| | |
|---|---|
| Reporting on sex and gender | We used female blood only as they are breast cancer patients |
| Reporting on race, ethnicity, or other socially relevant groupings | No race-, ethnicity-, and other socially relevant groupings-based analyses were conducted. We collected blood samples from patients based mainly on the diagnosis of metastatic breast cancer during the study period. |
| Population characteristics | Current diagnosis |
| Recruitment | Outpatient visit and patient consent |
| Ethics oversight | Dana-Farber Cancer Institute |

Note that full information on the approval of the study protocol must also be provided in the manuscript.

# Field-specific reporting

Please select the one below that is the best fit for your research. If you are not sure, read the appropriate sections before making your selection.

☒ Life sciences ☐ Behavioural & social sciences ☐ Ecological, evolutionary & environmental sciences

For a reference copy of the document with all sections, see nature.com/documents/nr-reporting-summary-flat.pdf

# Life sciences study design

All studies must disclose on these points even when the disclosure is negative.

| | |
|---|---|
| Sample size | In vitro experiments were performed using at least three technical replicates and each experiment was repeated independently at least three times. Sample sizes for in vitro experiments were chosen based on the standard in the field. From pilot data, we estimate the mean of tumor size in the CAR T cells treated group is around 50.4 with SD=3.2 mm3 and the mean of tumor size and SD in the DSF/Cu+IR+CAR T cells treated group are around zero. With a sample size of 5 per group, we have >90% power to detect this difference using a two-sided t-test at the 0.05 alpha level. |

| | |
|---|---|
| Data exclusions | No data was excluded from the analysis. |
| Replication | All in vitro experiments were performed independently for at least 3 times. All in vivo experiments were conducted once and further validated using several mouse tumor models for corroboration. |
| Randomization | For in vivo studies, the mice were divided into groups using a stratified randomization strategy. |
| Blinding | Prior to treatment, mice were randomized to ensure similar average tumor sizes among all experimental groups. To meet the requirements of our animal care facility, detailed cage labeling was implemented, and investigators were not blinded to group allocation. To minimize potential bias, every effort was made to process and analyze all samples uniformly, ensuring consistency across different groups. Specific measures were taken to avoid any study bias for in vitro experiments. While blinding was not employed, experiments were meticulously organized into groups, including relevant controls, and the analysis was conducted objectively. |

# Reporting for specific materials, systems and methods

We require information from authors about some types of materials, experimental systems and methods used in many studies. Here, indicate whether each material, system or method listed is relevant to your study. If you are not sure if a list item applies to your research, read the appropriate section before selecting a response.

## Materials & experimental systems

| n/a | Involved in the study |
|---|---|
| ☐ | ☒ Antibodies |
| ☐ | ☒ Eukaryotic cell lines |
| ☒ | ☐ Palaeontology and archaeology |
| ☐ | ☒ Animals and other organisms |
| ☒ | ☐ Clinical data |
| ☒ | ☐ Dual use research of concern |
| ☒ | ☐ Plants |

## Methods

| n/a | Involved in the study |
|---|---|
| ☒ | ☐ ChIP-seq |
| ☐ | ☒ Flow cytometry |
| ☒ | ☐ MRI-based neuroimaging |

## Antibodies

| | |
|---|---|
| Antibodies used | These antibodies were used for flow cytometry: R-Phycoerythrin AffiniPure F(ab')$_2$ Fragment Goat Anti-Mouse IgG (H+L) (cat#115-116-146, 1:100), Allophycocyanin (APC) AffiniPure F(ab')$_2$Fragment Goat Anti-Mouse IgG (H+L) (cat#115-136-146, 1:100), Fluorescein (FITC) AffiniPure F(ab')$_2$ Fragment Goat Anti-Mouse IgG (H+L) (cat# 115-096-146, 1:100) were obtained from Jackson ImmunoResearch. CD3-PE-Cy7 (cat#300420, clone: UCHT1, 1:100), CD8-APC-Cy7 (cat#344714, clone: SK1, 1:100), CD4-FITC (cat#357406, clone: RM4-5, 1:100), CD45RA- PE/Cyanine5 (cat#304110, clone: HI100, 1:100), CD45RO-APC (cat#304210, clone: UCHL1, 1:100), CD62L-PE (cat#304806, clone: MEL-14, 1:100), CD27-BV421 (cat#302824, clone: O323, 1:100), CD45RA-FITC (cat#304148, clone: HI100, 1:100), CD45RA-BV421 (cat#304130, clone: HI100, 1:100), PD-1 (CD279) -APC (cat#329907, clone: EH12.2H7, 1:100), PD-1 (CD279)-PE (cat#621608, clone: A17188B, 1:100), LAG-3 (CD223)-APC (cat#369212, clone: 7H2C65, 1:100), CCR7 (CD197) -BV421 (cat#353208, clone: G043H7, 1:100), CD261 (DR4, TRAIL-R1)-PE (cat#307206, clone: DJR1, 1:100), CD262 (DR5, TRAIL-R2)-APC (cat#307408, clone: DJR2-4 (7-8) , 1:100), CD95 (Fas)-BV785 (cat#305646, clone: DX2, 1:100), CD127 (IL-7Rα)-PE (cat#351304, clone: A019D5, 1:100), TNFα-PE (cat#502909, clone: Mab11, 1:100), IFNγ-APC (cat#502512, clone: 4S.B3, 1:100), Granzyme B-Pacific Blue (cat#515408, clone: GB11, 1:100), CD62L-BV711 (cat#304860, clone: DREG-56, 1:100), CD253 (TRAIL)-PE (cat#308206, clone: RIK-2, 1:100), CD45-PE (cat#368510, clone: 2D1, 1:100), CD366 (TIM3)-Pacific Blue (cat#345042, clone: F38-2E2, 1:100), CD279 (PD-1)-PerCP/Cyanine5.5 (cat#367410, clone: NAT105, 1:100), CD152 (CTLA-4)-BV605 (cat#369610, clone: BNI3, 1:100), CD25-PE/Dazzle™ 594 (cat#302646, clone: BC96, 1:100), CD276 (B7-H3)-APC (cat#351005, clone: MIH42, 1:100), CD11b-BV510 (cat#301334, clone: ICRF44, 1:100), CD14-APC/Cyanine7 (cat#325620, clone: HCD14, 1:100), CD33-PE/Cyanine7 (cat#366618, clone: P67.6, 1:100), CD86- PE/Cyanine7 (cat#374209, clone: BU63, 1:100), CD56-APC (cat#362503, clone: 5.1H11, 1:100), CD20-BV510 (cat#302339, clone: 2H7, 1:100), HLA-DR-APC (cat# 307609, clone: L243, 1:100), and Zombie Red™ Fixable Viability Kit (cat# 423110) were purchased from Biolegend. CD3-FITC (cat#555339, clone: HIT3a, 1:100), and FITC-Labeled Human B7-H3 (4Ig) / B7-H3b Protein (cat# B7B-HF2E7-25μg, 0.5μg/100μL) were obtained from BD Biosciences and Acro Biosytems, respectively. These antibodies were used for western blot:ATF-6 (cat#65880S, 1:1000), Phospho-eIF2α (Ser51) (cat#3398, 1:1000), Bcl-2 (cat#15071S, 1:1000), β-Actin (#3700, 1:2000), STAT5 (cat#25656S, 1:1000), Phospho-Stat5 (Tyr694) (cat#9356S, 1:1000), STAT3 (cat#9139S, 1:1000), JAK1 (cat#3344S, 1:1000), Phospho-Jak2 (Tyr1007/1008) (cat#3771, 1:1000), Phospho-Stat3 (Tyr705) (cat#9145, 1:1000), Phospho-Jak1 (Tyr1034/1035) (cat#74129, 1:1000) and Jak2 (cat#3230, 1:1000) were obtained from Cell Signaling Technology. Phospho-IRE1 alpha (Ser724) (cat# PA585647, 1:1000) were obtained from Invitrogen. These antibodies were used for CAR T generation: CD28 (cat#556620, clone: CD28.2, 1μg/mL) were purchased from Biosciences, and CD3 (cat#130-093-387, clone: OKT3, 1μg/mL) from Miltenyi Biotec. |
| Validation | All antibodies were used based on the validation statements on the manufacturer's website, including the specie and applications. |

## Eukaryotic cell lines

Policy information about cell lines and Sex and Gender in Research

| | |
|---|---|
| Cell line source(s) | Human TNBC cell lines, SUM149 and SUM159, were acquired from the Duke Comprehensive Cancer Center Cell Culture Facility and Asterand Bioscience, Inc., respectively. The human pancreatic ductal adenocarcinoma (PDAC) cell line PANC-1 |

| | |
|---|---|
| | was purchased from American Type Culture Collection (ATCC). The human PDAC cell line PDAC-6 was established at MGH using ascites fluid from a patient with metastatic PDAC. The head and neck squamous cell carcinoma (HNSCC) cell line PCI-13 was obtained from Dr. Theresa L. Whiteside at UPMC Hillman Cancer Center, the University of Pittsburgh. |
| Authentication | All cell lines were authenticated by providers. |
| Mycoplasma contamination | All cell lines were tested negative for mycoplasma contamination. |
| Commonly misidentified lines (See ICLAC register) | No commonly misidentified cell lines was used in the study. |

## Animals and other research organisms

Policy information about studies involving animals; ARRIVE guidelines recommended for reporting animal research, and Sex and Gender in Research

| | |
|---|---|
| Laboratory animals | The 6-to-8-week-old NSG mice were obtained from the Massachusetts General Hospital (MGH) COX7 animal facility or The Jackson laboratory. NSG mice were housed in autoclaved polysulfone individually ventilated cages (Allentown Caging, Allentown, NJ) in a specific pathogen-free (SPF) environment. Room lights were maintained on a 12:12-hour light: dark cycle. Room temperature was maintained at 68 to 71 F, and room humidity remained between 30% and 60%. |
| Wild animals | No wild animals were used in the study. |
| Reporting on sex | We utilized both female and male mice in our study, as determined by mouse tumor models. |
| Field-collected samples | No field collected samples were used in the study. |
| Ethics oversight | The MGH Institutional Animal Care and Use Committee approved the animal studies described herein. |

Note that full information on the approval of the study protocol must also be provided in the manuscript.

## Flow Cytometry

### Plots

Confirm that:

- [x] The axis labels state the marker and fluorochrome used (e.g. CD4-FITC).
- [x] The axis scales are clearly visible. Include numbers along axes only for bottom left plot of group (a 'group' is an analysis of identical markers).
- [x] All plots are contour plots with outliers or pseudocolor plots.
- [x] A numerical value for number of cells or percentage (with statistics) is provided.

### Methodology

| | |
|---|---|
| Sample preparation | To measure the number of CAR T cells that had infiltrated tumor tissues and spleens, the tumor samples were digested with collagenase IV (0.5 mg/ml; Sigma-Aldrich) and deoxyribonuclease (DNase) I (0.2 mg/ml; Sigma-Aldrich) for 30 min at 37°C. Tumor digests and spleens were filtered through 70 µm cell strainers to obtain a single-cell suspension. ACK lysing buffer (Themo Fisher) was used to lyse the red blood cells according to the manufacturer's instructions. |
| Instrument | BD LSR II and BD Accuri C6. |
| Software | BD FACSDiva software (VERSION 8.0), BD Accuri C6 software (VERSION 1.0.264.21) and FlowJo software (VERSION 10.8.1, Ashland, OR). |
| Cell population abundance | By combining information from forward scatter (FSC), side scatter (SSC), and fluorescence intensity measurements, flow cytometry can distinguish and quantify different cell populations within a sample. |
| Gating strategy | We provided our exemplifying gating strategy in Supplementary Figure 8. Gating strategy: cells were gated on forward (FSC-A) and side (SSC-A) scatter, followed by gating on single cells (FSC-A, FSC-W and SSC-A, SSC-W) and live cell population based on Viability Dyes staining. Live cells were gated on anti-human CD3, CD4, CD8 for T cell population; anti-human CD56 for natural killing cells; anti-human CD20 for B cells; anti-human CD14 for monocytes and anti-human CD14, CD11b, CD86, HLA-DR for dendritic cells (DCs). |

- [x] Tick this box to confirm that a figure exemplifying the gating strategy is provided in the Supplementary Information.
